# Design, Synthesis, and Insecticidal Activity of Some Novel Diacylhydrazine and Acylhydrazone Derivatives

**DOI:** 10.3390/molecules20045625

**Published:** 2015-03-30

**Authors:** Jialong Sun, Yuanming Zhou

**Affiliations:** College of Chemistry and Pharmaceutical Sciences, Qingdao Agricultural University, Qingdao 266109, China; E-Mail: sunjialong6289@163.com

**Keywords:** diacylhydrazine, acylhydrazone, aromatic diamide, insecticidal activity, synthesis

## Abstract

In this study a series of diacylhydrazine and acylhydrazone derivatives were designed and synthesized according to the method of active group combination and the principles of aromatic group bioisosterism. The structures of the novel derivatives were determined on the basis on ^1^H-NMR, IR and ESI-MS spectral data. All of the compounds were evaluated for their *in vivo* insecticidal activity against the third instar larvae of *Spodoptera* *exigua* Hiibner, *Helicoverpa* *armigera* Hubner, *Plutella* *xyllostella* Linnaeus and *Pieris* *rapae* Linne, respectively, at a concentration of 10 mg/L. The results showed that all of the derivatives displayed high insecticidal activity. Most of the compounds presented higher insecticidal activity against *S. exigua* than the reference compounds tebufenozide, metaflumizone and tolfenpyrad, and approximately identical insecticidal activity against *H.* *armigera*, *P.* *xyllostella* and *P.* *rapae* as the references metaflumizone and tolfenpyrad.

## 1. Introduction

Synthetic pesticides have performed major functions in feed, food and fiber production for many years. To a considerable extent, these pesticides may be expected to be used well into the future, although the type of pesticides used may shift toward materials with novel modes of action and lower risk to humans and other non-target organisms [1,2].

**Figure 1:**
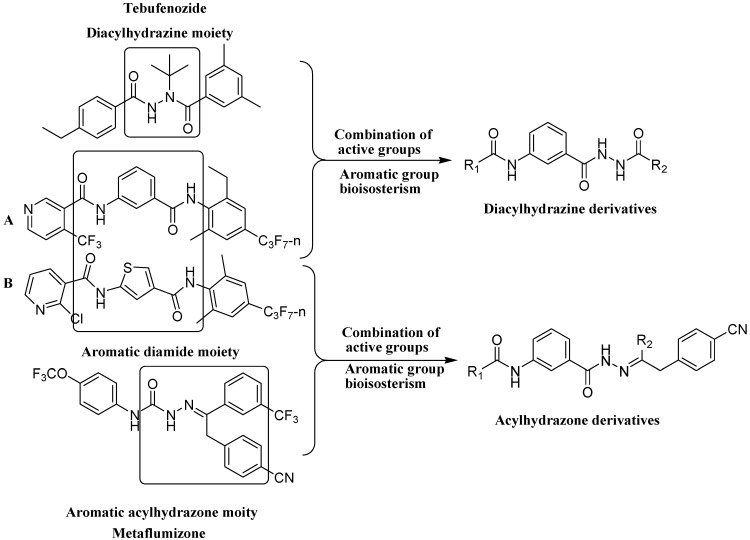
Design of the skeleton of diacylhydrazine and acylhydrazone derivatives.

The phthalic diamide flubendiamide and the anthranilic diamides chlorantraniliprole and cyantraniliprole were successively designed and synthesized by Japanese pesticide companies in 1998 and DuPont in 2001; these novel insecticides act on the ryanodine receptor (RyR) [3,4,5,6,7]. Bayer and Syngenta have also developed the *meta*-amino benzamides A and B (Figure 1), both of which are considered RyR pesticides [8,9,10]. Compounds A and B present excellent insecticidal activities against pests of different orders, such as Lepidoptera, Diptera, Coleoptera, Hemiptera, and Isoptera, and feature high selectivity, low mammalian toxicity, and environmental friendliness. As determined through electrophysiological and Ca^2+^-release studies these insecticides act by activating insect RyR, a non-voltage-gated calcium channel, to affect calcium release from intracellular stores by locking channels in a partially opened state [3,11,12]. All of the RyR insecticides described above contain two amide structures that are important to their insecticidal activity.

Diacylhydrazines have been identified as one of the most important types of insect regulators since the discovery of the *N*-*tert*-butyl-*N*,*N*'-diacylhydrazines in the mid-1980s by Rohm and Haas [13,14,15]. Several commercial compounds, such as tebufenozide, methoxyfenozide, chromafenozide, and halofenozide, are all classified as diacylhydrazines, and all of these insecticides affect the ecdysone receptor complex, leading to precocious lethal molting, especially in caterpillars [16,17]. Diacylhydrazines have attracted significant attention because of their high insecticidal selectivity, simple structure, and low toxicity to vertebrates [15].

Metaflumizone, a semicarbazone compound with a structure containing an acylhydrazone moiety, is a novel sodium channel blocker insecticide recently introduced to the Chinese market in 2010 by BASF. It provides excellent control of most economically important pests belonging to the orders Lepidoptera, Coleoptera, Hymenoptera, Hemiptera, Isoptera, Diptera, and Siphonaptera. Metaflumizone presents low risk to pollinators and beneficial insects, as well as humans and the environment. Insect strains that are resistant to organophosphates, carbamates, and imidacloprid do not display cross-resistance to metaflumizone. Thus, this insecticide demonstrates great potential use in Integrated Pest Management (IPM) and resistance management strategies [2,18,19]. Moreover many other acylhydrazone compounds also show strong insecticidal activity [20,21].

Plant pests, such as the beet armyworm (*Spodoptera* *exigua* Hiibner), diamondback moth (*Helicoverpa* *armigera* Hubner), cotton bollworm (*Plutella* *xyllostella* Linnaeus), and cabbage caterpillar (*Pieris* *rapae* Linne), are harmful to crops all over the world. Unfortunately, pest control of these species has become increasingly difficult because of development of resistance to traditional insecticides. Poor pest control leads to enormous losses of crop production because of long-term use of conventional pesticides [3,22].

**Scheme 1:**
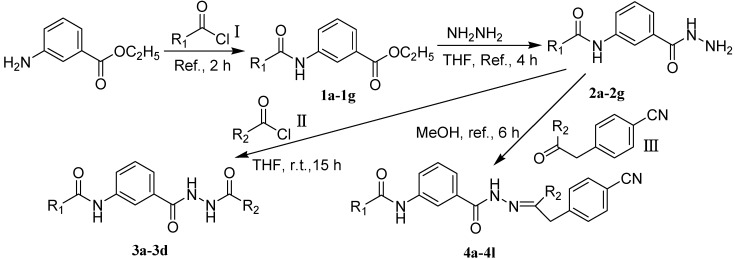
Synthesis of diacylhydrazine and acylhydrazone derivatives.

In the present work, we sought to incorporate an aromatic diamide unit into an aromatic diacylhydrazine or acylhydrazone moiety according to the method of active group combination and the principle of aromatic group bioisosterism. A total of four diacylhydrazine derivatives and 12 acylhydrazone derivatives were designed; the structures of these compounds combined a *meta*-amino benzamide with diacylhydrazine or acylhydrazone active groups (Scheme 1) and they were identified by IR, ESI-MS, and ^1^H-NMR spectroscopy. The insecticidal activities of the resulting compounds against the third instarlarvae of beet armyworm, diamondback moth, cotton bollworm, and cabbage caterpillar were screened. Combination of critical components is expected to improve the biological activities of these pesticides.

## 2. Results and Discussion

### 2.1. Chemistry

Scheme 1 shows the route used successfully for the preparation of the four diacylhydrazine derivatives **3a**–**3d** and 12 acylhydrazone derivatives **4a**–**4l**. The raw materials 3-aminoethyl benzoate and acyl chlorides R_1_COCl (**I**) were dissolved in chloroform and heated to reflux to prepare the 3-acylamino ethyl benzoates **1a**–**1g**. Using alcohol as a solvent, 3-acylaminobenzoyl hydrazines **2a**–**2g** were obtained by reaction of compounds **1a**–**1g** with hydrazine hydrate. Then, compounds **2a**–**2g** were reacted with acyl chlorides R_2_COCl (**II**) in tetrahydrofuran at room temperature to prepare the diacylhydrazine derivatives **3a**–**3d**. Again using alcohol as a solvent and trifluoroacetic acid as a catalyst, compounds **2a**–**2g** were reacted with 2-(4-nitrilo)benzyl-1-substituedphenyl ketones **II** to form acylhydrazone derivatives **4a**–**4l**. The structures of all of the diacylhydrazine and acylhydrazone derivatives were effectively determined through ^1^H-NMR, ESI-MS, and IR spectroscopy.

### 2.2. Insecticidal Activities

Table 1 shows that all of the diacylhydrazine and acylhydrazone derivatives **3a**–**3d**, **4a**–**4l** display strong insecticidal activity against the third instar larvae of beet armyworm (*S. exigua*) at a concentration of 10 mg/L. Most of the synthesized compounds indicated higher insecticidal activity than the reference compounds tebufenozide, metaflumizone, and tolfenpyrad. Among the synthesized compounds, the mortality caused by compounds **3b**, **4b**, **4c**, **4d**, **4f** and **4l** exceeded 95% at the 72 h time point. Moreover the third instar larvae of beet armyworm showed 100% mortality within 72 h when treated with compounds **4b**, **4d**, and **4l**. Table 1 further demonstrates that insect mortality presents a positive relationship with administration time.

Table 2 presents the mortality data of cotton bollworm (*H.* *armigera*), diamondback moth (*P. xyllostella*), and cabbage worm (*P. rapae*) exposed to acylhydrazone derivatives **4a**–**4l** at a concentration of 10 mg/L for 72 h. Compounds **4a**–**4l** revealed strong insecticidal activity against the third instar larvae of these species. Insect mortalities from exposure to these compounds were approximately identical to those observed from exposure to the reference compounds metaflumizone and tolfenpyrad. Among the synthesized acylhydrazone derivatives, **4b**, **4c**, **4d**, **4f** and **4l** showed 100% mortalities against the third instar larvae of *P.* *rapae*. Compound **4f** in particular displayed broad spectrum insecticidal activity.

According to the data in Table 1 and Table 2, it could be found that the presence of fluorine was important for the insecticidal activity of the synthesized compounds. Comparing the differences between the substituent groups and the position of the substituents on the benzene ring, it could be presumed that relatively weak electron-withdrawing effects could strengthen the insecticidal activities of the acylhydrazone derivatives, however electron-donating effects (such as seen in **4j**) and strong electron-withdrawing effects (such as in **4h** and **4i**) could not do so.

**Table 1:** Insecticidal activities of diacylhydrazine and acylhydrazone derivatives (concentration, 10 mg/L) against the third instar larvae of beet armyworm.

Compound	R_1_	R_2_	Mortality (%)		
			24 h	48 h	72 h
**3a**	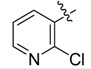	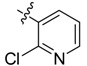	12.50	29.17	70.83
**3b**	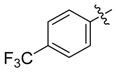	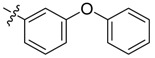	45.83	66.67	95.83
**3c**	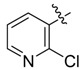	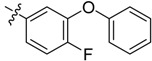	16.67	37.50	79.17
**3d**	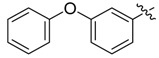	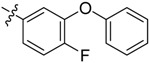	41.67	66.67	91.67
**4a**	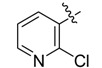	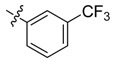	45.83	66.67	87.50
**4b**	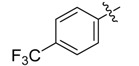	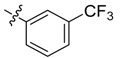	50.00	75.00	100.00
**4c**	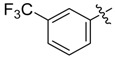	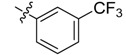	50.00	79.17	95.83
**4d**	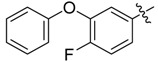	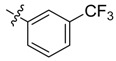	54.17	79.17	100.00
**4e**	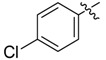	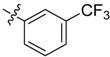	45.83	66.67	91.67
**4f**	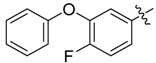	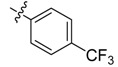	54.17	70.83	95.83
**4g**	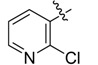	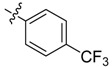	41.67	70.83	91.67
**4h**	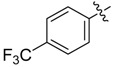	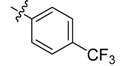	37.50	75.00	91.67
**4i**	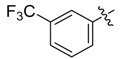	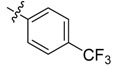	45.83	70.83	91.67
**4j**	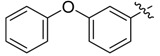	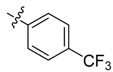	37.50	66.67	87.50
**4k**	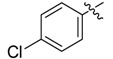	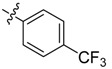	41.67	70.83	87.50
**4l**	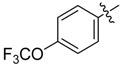	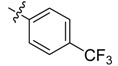	58.33	83.33	100.00
**Reference Compounds**					
**Tebufenozide**			12.50	37.50	66.67
**Metaflumizone**			37.50	45.83	66.67
**Tolfenpyrad**			45.83	66.67	79.17

**Table 2:** Insecticidal activities of acylhydrazone derivatives (concentration, 10 mg/L; treatment time, 72 h).

Compound	Mortality(%)		
	*H. armigera*	*P. xyllostella*	*P. rapae*
**4a**	87.50	79.17	91.67
**4b**	91.67	95.83	100.0
**4c**	91.67	87.50	100.0
**4d**	95.83	87.50	100.0
**4e**	79.17	83.33	91.67
**4f**	95.83	100.0	100.0
**4g**	87.50	87.50	95.83
**4h**	83.33	87.50	95.83
**4i**	87.50	75.50	91.67
**4j**	83.33	83.33	91.67
**4k**	91.67	70.83	91.67
**4l**	79.17	95.83	100.0
**Metaflumizone**	87.50	83.33	87.50
**Tolfenpyrad**	91.67	91.67	95.83

## 3. Experimental Section 

### 3.1. General Procedures

All reagents were chemically pure and solvents were dried according to standard methods. ^1^H-NMR spectra were obtained on an AM-500 spectrometer (Bruker, Karlsruhe, Germany) with DMSO-*d_6_* as the solvent. IR spectra were recorded on an IR-200 spectrophotometer (Nicolet, Thermo Electron, Madison, WI, USA) using KBr disks. Mass spectra were recorded under ESI conditions on a Q-TOF spectrometer (Micromass, Waters Corp., Manchester, UK). Melting points were measured on a WRS-1A-type melting point apparatus (Shanghai, China) and are reported uncorrected. Analytical TLC was carried out on pre-coated silica gel plates, and spots were visualized through UV illumination (254 nm).

### 3.2. General Procedure for the Preparation of **1a**–**1g**

3-Aminoethyl benzoate (30 mmol) was dissolved in chloroform (25 mL) in an ice-water bath. Then, an acyl chloride R_1_COCl (**I**, 30 mmol) was dissolved in chloroform (25 mL) and added dropwise to this solution. The mixture was reacted at 25 °C in a water bath for 5 h and then refluxed for 2 h. The reaction mixture was subsequently cooled to room temperature and filtered under vacuum to obtain the 3-acylaminoethyl benzoates **1a**–**1g** with yields of 85%–95%.

### 3.3. General Procedure for the Preparation of **2a**–**2g**

A mixture of 3-acylaminoethyl benzoates **1a**–**1g** (20 mmol), 80% hydrazine hydrate (100 mmol), and ethanol (100 mL) was stirred and heated under reflux for 4 h. The reaction mixture was cooled to room temperature and filtered under vacuum. The solid obtained was washed with water (50 mL) to provide the 3-acylaminobenzoyl hydrazines **2a**–**2g** in yields of 80%–95%.

### 3.4. General Procedure for Preparation of **3a**–**3d** and **4a**–**4l**

3-Acylaminobenzoyl hydrazines **2a**–**2g** (5 mmol) and NaOH (5.7 mmol) were dissolved in dry tetrahydrofuran (50 mL). Acyl chlorides R_2_COCl (**II**, 5.7 mmol) dissolved in dry tetrahydrofuran (30 mL) were then dropped slowly into the above solution at 0 °C in an ice-water bath. The solution was reacted at room temperature for 15 h. After completion of the reaction, the reaction mixture was concentrated *in vacuo* and mixed with saturated NaHCO_3_ solution (25 mL). The mixture was stirred for 30 min and then filtered under vacuum. The filtrate was recrystallized with DMF and water (volume ratio, 2:1) to afford the target compounds **3a**–**3d**.

3-Acylaminobenzoyl hydrazines **2a**–**2g** (2 mmol**)**, 2-(4-nitrilo)benzyl-1-substituted phenyl ketones (**III**, 2 mmol), and two or three drops of trifluoroacetic acid were dissolved in methanol (15 mL) and *n-*hexane (2 mL). This solution was stirred and heated under reflux for 6 h, after which the reaction mixture was concentrated *in vacuo* and filtered under vacuum. The filtrate was recrystallized with methanol to yield the target compounds **4a**–**4l**. All 16 compounds are novel compounds and their physical and spectral data are listed below.

*N-(2-Chloropyridyl-3-formyl)-3-(2-chloropyridyl-3-formylamino)benzoyl hydrazine* (**3a**). White needle-like crystals, yield 75.2%, m.p. 253.0–253.2 °C. HR-ESI-MS *m/z*: 430.0471 [M+H]^+^ (calcd for C_19_H_14_Cl_2_N_5_O_3_, 430.0468). ^1^H-NMR (DMSO-*d*_6_) δ: 10.84, 10.73, 10.61 (each s, 1H, NH), 8.54 (d, *J* = 2.4Hz, 2H,), 8.26 (s, 1H), 8.10 (dd, *J* = 7.5, 1.7 Hz, 1H), 7.98 (dd, *J* = 7.5, 1.7 Hz, 1H), 7.87 (d, *J* = 8.0 Hz, 1H), 7.68 (d, *J* = 7.8 Hz, 1H), 7.60–7.48 (m, overlapped, 3H). IR (KBr): *ν* 3246, 2360, 1701, 1656, 1580, 1492, 1400, 1283, 1268, 912, 750, 653 cm^−1^.

*N-(3-Phenoxy)benzoyl-3-(3-phenoxylbenzoylamino)benzoyl* *hydrazine* (**3b**). White powder, yield 76.6%, m.p. 239.6–240.8 °C. HR-ESI-MS *m/z*: 520.1474 [M+H]^+^ (calcd for C_28_H_21_F_3_N_3_O_4_, 520.1479). ^1^H-NMR (DMSO-*d*_6_) δ: 10.67, 10.57, 10.52 (each s, 1H, NH), 8.30 (s, 1H), 8.18 (d, *J* = 8.1 Hz, 2H), 8.01 (d, *J* = 8.1 Hz, 1H), 7.93 (d, *J* = 8.2 Hz, 2H), 7.71 (d, *J* = 7.7 Hz, 1H), 7.66 (d, *J* = 7.7 Hz, 1H), 7.50–7.54 (m, overlapped, 3H), 7.43 (t, *J* = 7.8 Hz, 2H), 7.25 (d, *J* = 8.1 Hz, 1H), 7.19 (t, *J* = 7.4 Hz, 1H), 7.08 (d, *J* = 8.1 Hz, 2H).

*N-(4-Fluoro-3-phenoxy)benzoyl-3-(2-chloropyridyl-3-formylamino)benzoyl hydrazine* (**3c**). White powder, yield 78.3%, m.p. 185.6–186.4 °C. ^1^H-NMR (DMSO-*d*_6_) δ: 10.89, 10.60, 10.46 (each s, 1H, NH), 8.55 (dd, *J* = 4.0, 1.6 Hz, 1H), 8.28 (s, 1H), 7.99 (dd, *J* = 6.2, 1.6 Hz, 1H), 7.95 (dd, *J* = 6.7, 1.0 Hz, 1H), 7.92 (m, 1H), 7.82 (dd, *J* = 6.5, 1.8 Hz, 1H), 7.66 (d, *J* = 6.6 Hz, 1H), 7.56–7.61 (m, overlapped, 2H), 7.49 (t, *J* = 6.6 Hz, 1H), 7.42 (dd, *J* = 7.1, 6.6 Hz, 2H),7.17 (t, *J* = 6.1 Hz, 1H), 7.06 (d, *J* = 6.5 Hz, 2H). IR (KBr): *ν* 3478, 3411, 1704, 1615, 1577, 1392, 1388, 1164, 902, 714 cm^−1^.

*N-(4-Fluoro-3-phenoxy)benzoyl-3-(3-phenoxybenzoylamino)benzoyl hydrazine* (**3d**). White powder, yield 72.6%, m.p. 221.8–222.1 °C. HR-ESI-MS *m/z*: 562.1777 [M+H]^+^ (calcd for C_33_H_25_FN_3_O_5_, 562.1773). ^1^H-NMR (DMSO-*d*_6_) δ: 10.60, 10.51, 10.47 (each s, 1H, NH), 8.55 (s, 1H), 8.26 (s, 1H), 7.99 (d, *J* = 7.6 Hz, 1H), 7.84–7.76 (m, overlapped, 2H), 7.67 (d, *J* = 7.9 Hz, 1H), 7.59 (m, 4H), 7.40–7.50 (m, overlapped, 5H), 7.24 (d, *J* = 8.1 Hz, 1H), 7.19 (t, *J* = 7.4 Hz, 2H), 7.08 (d, *J* = 8.1 Hz, 3H). IR (KBr): *ν* 3296, 3222, 3072, 1648, 1612, 1580, 1545, 1477, 1274, 900, 685 cm^−1^.

*N-[3-(2-Chloropyridyl-3-formylamino)]benzoyl-1-(3-trifluoromethyl)phenyl-2-(4-cyano)phenyl ethanone* *hydrazone* (**4a**). White needle-like crystals, yield 63.4%, m.p. 253.0–253.2 °C. HR-ESI-MS *m/z*: 562.1256 [M+H]^+^ (calcd for C_29_H_20_ClF_3_N_5_O_2_, 562.1252). ^1^H-NMR (DMSO-*d*_6_) δ: 11.39, 10.86 (each s, 1H, NH), 8.55 (dd, *J* = 4.8,1.7 Hz, 1H), 8.16 (d, *J* = 6.2 Hz, 1H), 8.10 (dd, *J* = 7.5, 1.6 Hz, 1H), 8.03 (s, 1H), 7.92 (d, *J* = 8.2 Hz, 1H), 7.88 (d, *J* = 6.0 Hz, 1H), 7.72–7.82 (overlapped, 4H), 7.58(dd, *J* = 7.5, 4.9 Hz, 1H), 7.52 (s, 1H), 7.51 (d, *J* = 7.1 Hz, 1H), 7.40 (d, *J* = 8.1 Hz, 2H), 4.58 (s, 2H, -CH_2_-). IR (KBr): *ν* 3275, 2230,1671, 1550, 1400, 1309,1103, 1068, 841, 750 cm^−1^.

*N-[3-(4-Trifluoromethyl)benzoylamino]benzoyl-1-(3-trifluoromethyl)phenyl-2-(4-cyano)phenyl ethanone* *hydrazone* (**4b**). Off-white powder, yield 68.6%, m.p. 217.9–218.5 °C. ^1^H-NMR (DMSO-*d*_6_) δ: 11.35, 10.65 (each s, 1H, NH), 8.16 (d, *J* = 7.8 Hz, 3H), 7.99 (s,1H), 7.93 (s, 1H), 7.92 (d, *J* =8.8 Hz, 3H), 7.76 (d, *J* = 8.0 Hz, 2H), 7.60 (s, 1H), 7.50 (s, 1H), 7.49 (d, *J* = 7.4 Hz, 1H), 7.39 (d, *J* = 8.0 Hz, 2H), 4.58 (s, 2H, -CH_2_-). IR (KBr): *ν* 3222, 2360, 2227, 1651, 1550, 1321, 1162, 862, 685 cm^−1^.

*N-[3-(3-Trifluoromethyl)benzoylamino]benzoyl-1-(3-trifluoromethyl)phenyl-2-(4-cyano)phenyl ethanone* *hydrazone* (**4c**). Off-white powder, yield 77.9%, m.p. 184.0–184.5 °C. HR-ESI-MS *m/z*: 595.1560 [M+H]^+^ (calcd for C_31_H_21_F_6_N_4_O_2_, 595.1563). ^1^H-NMR (DMSO-*d*_6_) δ: 11.36, 10.67 (each s, 1H, NH), 8.33 (s, 1H), 8.29 (d, *J* = 7.9 Hz, 1H), 8.23 (s, 1H), 8.03 (overlapped, 2H), 7.99 (d, *J* = 7.9 Hz, 1H), 7.55–7.82 (m, overlapped, 5H), 7.52 (s, 1H), 7.51 (d, *J* = 6.7 Hz, 1H),7.39 (d, *J* = 8.2 Hz, 2H), 4.59 (s, 2H, -CH_2_-). IR (KBr): *ν* 3225, 2357, 2227, 1680, 1651, 1550, 1336,1108, 853, 697 cm^−1^.

*N-(4-Fluoro-3-phenoxylbenzoylamino)benzoyl-1-(3-trifluoromethyl)phenyl-2-(4-cyano)phenyl ethanone* *hydrazone* (**4d**). Off-white powder, yield 74.3%, m.p.196.3–196.8 °C. HR-ESI-MS *m/z*: 637.1858 [M+H]^+^ (calcd for C_36_H_25_F_4_N_4_O_3_, 637.1857). ^1^H-NMR (DMSO-*d*_6_) δ: 11.34, 10.46 (each s, 1H, NH), 8.18 (s, 1H), 8.08 (s, 1H), 7.88–7.98 (m, 3H), 7.65–7.85 (m, 4H), 7.58–7.70 (m, 2H), 7.48 (s, 1H), 7.36–7.47 (m, 3H), 7.18 (m, 1H), 7.06 (d, *J* = 7.3 Hz, 2H), 4.59 (s, 2H, -CH_2_-). IR (KBr): *ν* 2360, 2227, 1671, 1648, 1559, 1333, 1277, 1212, 1135, 876, 750 cm^−1^.

*N-(4-Chlorobenzoylamino)]benzoyl-1-(3-trifluoromethyl)phenyl-2-(4-cyano)phenyl ethanone* *hydrazone* (**4e**). White powder, yield 70.3%, m.p. 197.0–197.3 °C. HR-ESI-MS *m/z*: 561.1298 [M+H]^+^ (calcd for C_30_H_21_ClF_3_N_4_O_2_, 561.1300). ^1^H-NMR (DMSO-*d*_6_) δ: 11.35, 10.52 (each s, 1H, NH), 8.23 (s, 1H), 8.17 (s, 1H), 8.01 (d, *J* = 8.4 Hz, 3H), 7.94 (s, 1H), 7.78 (d, *J* = 8.2 Hz, 2H), 7.63 (d, *J* = 8.5 Hz, 3H), 7.49 (s, 1H), 7.48 (d, *J* = 6.7 Hz,1H), 7.40 (d, *J* = 8.2 Hz, 2H), 4.60 (s, 2H, -CH_2_-). IR (KBr): *ν* 3255, 2227, 1659, 1542, 1341, 1109, 1074, 826, 750 cm^−1^.

*N-(4-Fluoro-3-phenoxylbenzoylamino)benzoyl-1-(4-trifluoromethyl)phenyl-2-(4-cyano)phenyl ethanone* *hydrazone* (**4f**). Off-white powder, yield 74.3%, m.p. 187.1–187.4 °C. HR-ESI-MS *m/z*: 637.1859 [M+H]^+^ (calcd for C_36_H_25_F_4_N_4_O_3_, 637.1858). ^1^H-NMR (DMSO-*d*_6_) δ: 11.33, 10.45 (each s,1H, NH), 8.18 (s, 1H), 7.90–8.10 (m, 4H), 7.70–7.82 (m, 5H), 7.60 (m,1H), 7.35–7.50 (m, 6H), 7.18 (t, *J* = 7.3 Hz, 1H), 7.06 (d, *J* = 8.1 Hz, 2H), 4.57 (s, 2H, -CH_2_-). IR (KBr): *ν* 3187, 2369, 2225, 1677, 1648, 1556, 1503, 1324, 1271.3, 1212, 1132, 753, 691 cm^−1^.

*N-[3-(2-Chloropyridyl-3-formylamino)]benzoyl-1-(4-trifluoromethyl)phenyl-2-(4-cyano)phenyl ethanone* *hydrazone* (**4g**). White powder, yield 65.7%, m.p. 244.4–245.2 °C. HR-ESI-MS *m/z*: 562.1254 [M+H]^+^ (calcd for C_29_H_20_ClF_3_N_5_O_2_, 562.1252). ^1^H-NMR (DMSO-*d*_6_) δ: 11.39, 10.85 (each s, 1H, NH), 8.55 (dd, *J* = 4.4 Hz, 1H), 8.16 (s, 2H), 8.10 (m, 2H), 7.90 (d, *J* = 5.8 Hz, 1H), 7.77 (d, *J* = 8.0 Hz, 2H), 7.72 (d, *J* = 8.1 Hz, 1H), 7.62 (s, 1H), 7.58 (dd, *J* = 7.4, 4.9 Hz, 1H), 7.46–7.54 (m, 2H), 7.40 (d, *J* = 8.0 Hz, 2H), 4.59 (s, 2H, -CH_2_-). IR (KBr): *ν* 3275, 3249, 2227, 1671, 1527, 1397, 1350, 1168, 1103, 1068, 747, 547 cm^−1^.

*N-(4-Trifluoromethylbenzoylamino)benzoyl-1-(4-trifluoromethyl)phenyl-2-(4-cyano)phenyl ethanone* *hydrazone* (**4h**). Off-white powder, yield 78.3%, m.p. 228.7–229.0 °C. HR-ESI-MS *m/z*: 595.1568 [M+H]^+^ (calcd for C_31_H_21_F_6_N_4_O_2_, 595.1563). ^1^H-NMR (DMSO-*d*_6_) δ: 11.36, 10.67 (each s, 1H, NH), 8.24 (s, 1H), 8.18 (d, *J* = 8.0 Hz, 2H), 8.00 (s, 2H), 7.94 (d, *J* = 8.3 Hz, 2H), 7.70–7.82 (m, 5H), 7.49–7.55 (m, 2H), 7.40 (d, *J* = 8.3 Hz, 2H), 4.59 (s, 2H, -CH_2_-). IR (KBr): *ν* 3228, 2233, 1683, 1656, 1559, 1324, 1130, 1065, 853 cm^−1^.

*N-(3-Trifluoromethylbenzoylamino)benzoyl-1-(4-trifluoromethyl)phenyl-2-(4-cyano)phenyl ethanone* *hydrazone* (**4i**). White powder, yield 72.5%, m.p. 190.5–190.8 °C. HR-ESI-MS *m/z*: 595.1565 [M+H]^+^ (calcd for C_31_H_21_F_6_N_4_O_2_, 595.1563). ^1^H-NMR (DMSO-*d*_6_) δ: 11.35, 10.66 (each s, 1H, NH), 8.32 (s, 1H), 8.29 (d, *J* = 7.2 Hz, 2H), 8.23 (s, 1H), 8.18 (s, 1H), 7.96–8.05 (overlapped, 4H), 7.75–7.84 (m, overlapped, 4H), 7.63 (s, 1H), 7.52 (s, 1H), 7.51 (d, *J* = 7.3 Hz, 1H), 7.41 (d, *J* = 8.2 Hz, 2H), 4.60 (s, 2H, -CH_2_-). IR (KBr): *ν* 3267, 2230, 1659, 1512, 1336, 1142, 1074, 823, 553 cm^−1^.

*N-(3-Phenoxylbenzoylamino)benzoyl-1-(4-trifluoromethyl)phenyl-2-(4-cyano)phenyl ethanone* *hydrazone* (**4j**). Off-white powder, yield 70.3%, m.p. 189.4–189.6 °C. HR-ESI-MS *m/z*: 619.1949 [M+H]^+^ (calcd for C_36_H_26_F_3_N_4_O_3_, 619.1952). ^1^H-NMR (DMSO-*d*_6_) δ: 11.34, 10.47 (each s, 1H, NH), 8.22 (s, 1H), 7.95–8.10 (m, 3H), 7.71–7.82 (overlapped, 5H), 7.61 (s, 1H), 7.56 (t, *J* = 7.9 Hz, 1H), 7.48 (s, 1H), 7.47 (d, *J* = 6.5 Hz, 1H), 7.44 (t, *J* = 7.9 Hz, 2H), 7.39 (d, *J* = 8.1 Hz, 2H), 7.25 (dd, *J* = 8.0, 1.9 Hz, 1H), 7.19 (t, *J* = 7.5 Hz, 1H), 7.08 (d, *J* = 8.0 Hz, 2H), 4.58 (s, 2H,-CH_2_-). IR (KBr): *ν* 3187, 2225, 1677, 1648, 1539, 1480, 1327, 1271, 1235, 1124, 868, 750 cm^−1^.

*N-(4-Chlorobenzoylamino)]benzoyl-1-(4-trifluoromethyl)phenyl-2-(4-cyano)phenyl ethanone* *hydrazone* (**4k**). White powder, yield 70.3%, m.p. 208.5–208.7 °C. HR-ESI-MS *m/z*: 561.1304 [M+H]^+^ (calcd for C_30_H_21_ClF_3_N_4_O_2_, 561.1300). ^1^H-NMR (DMSO-*d*_6_) δ: 11.36, 10.52 (each s, 1H, NH), 8.23 (s, 1H), 7.95–8.10 (overlapped, 5H), 7.70–7.82 (overlapped, 4H), 7.63 (d, *J* = 8.5 Hz, 2H), 7.50 (s, 1H), 7.49 (d, *J* = 6.0 Hz, 1H), 7.40 (d, *J* = 8.0 Hz, 2H), 4.58 (s, 2H, -CH_2_-). IR (KBr): *ν*3225, 2233, 1680, 1645, 1559, 1486, 1327, 1127, 1065, 850, 753, 600 cm^−1^.

*N-(4-Trifluoromethoxy benzoylamino)]benzoyl-1-(4-trifluoromethyl) phenyl-2-(4-cyano) phenyl ethanone* *hydrazone* (**4l**). White powder, yield 70.3%, m.p. 249.5–249.8 °C. HR-ESI-MS *m/z*: 611.1518 [M+H]^+^ (calcd for C_31_H_21_F_6_N_4_O_3_, 611.1512). ^1^H-NMR (DMSO-*d*_6_) δ: 11.45, 10.58 (each s, 1H, NH), 8.35 (s, 1H), 8.19 (s, 1H), 8.16 (d, *J* = 7.9 Hz, 2H), 8.08 (s, 1H), 7.98 (d, *J* = 7.2 Hz, 1H), 7.91 (d, *J* = 9.0 Hz, 2H), 7.77 (d, *J* = 7.8 Hz, 2H), 7.68 (t, *J* = 7.7 Hz, 1H), 7.64 (s, 1H), 7.35–7.45 (m, overlapped, 4H), 4.61 (s, 2H, -CH_2_-). IR (KBr): *ν* 3222, 2230, 1683, 1645, 1536, 1509, 1256, 1171, 853, 697 cm^−1^.

### 3.5. Insecticidal Activity Bioassays

Wheat leaf discs measuring 0.5 cm × 0.5 cm were treated with 5 μL of 1.0 mg of test samples dissolved in 100 mL of acetone. Acetone was used as a negative control, whereas tebufenozide, metaflumizone, and tolfenpyrad were used as positive controls. The third instar larvae of *S.* *exigua* were allowed to feed on the discs. Cohorts of 24 beet armyworms were treated each time and bioassays were replicated three times. After 24, 48, and 72 h, the numbers of knocked-down larvae (indications: the larvae were narcotized, their bodies were very soft and immobile, and responses to stimuli disappeared completely) were recorded [23,24]. Insecticidal activity results are listed in Table 1.

Bioassays for insecticidal activity against cotton bollworm, diamondback moth, and cabbage worm was performed according to the method described above, except that the third instar larvae of *S.* *exigua* were replaced by the third instar larvae of *H. armigera*, *P. xyllostella*, and *P.* *rapae.* Metaflumizone and tolfenpyrad were used as positive controls, and the numbers of knocked-down larvae were recorded 72 h after exposure. Bioassay results are listed in Table 2.

## 4. Conclusions

In conclusion, four novel diacylhydrazine **3a**–**3d** and 12 acylhydrazone derivatives **4a**–**4l** were designed and synthesized according to the method of active group combination and the principle of aromatic group bioisosterism. The resultant analogs were evaluated (concentration, 10 mg/L) for their insecticidal activity against the third instar larvae of *S.* *exigua*, *H.* *armigera*, *P.* *xyllostella*, and *P.* *rapae in vivo*. Bioassays of these analogs showed high insecticidal activity. Most of the synthesized compounds presented higher insecticidal activity against *S. exigua* than the reference compounds tebufenozide, metaflumizone, and tolfenpyrad. Insecticidal activities against *H.* *armigera*, *P.* *xyllostella*, and *P.* *rapae* similar to that of the reference compounds metaflumizone and tolfenpyrad were also obtained. The results above motivate us to further explore novel diacylhydrazine and acylhydrazone derivatives as insecticidal agents; new findings will be reported in future work. 

## Supplementary Materials

Supplementary materials can be accessed at: http://www.mdpi.com/1420-3049/20/04/5625/s1.

## References

[1] Seiber J.N., Kleinschmidt L.A. (2011). Contribution of pesticide residue chemistry to improving food and environmental safety: past and present accomplishments and future challenges. J. Agric. Food Chem..

[2] Chatterjee N.S., Gupta S., Varghese E. (2013). Degradation of metaflumizone in soil: Impact of varying moisture, light, temperature, atmospheric CO_2_ level, soil type and soil sterilization. Chemosphere.

[3] Luo M., Chen Q.C., Wang J., Hu C.Y., Lu J., Luo X.M., Sun D.Q. (2014). Novel chlorantraniliprole derivatives as potential insecticides and probe to chlorantraniliprole binding site on ryanodine receptor. Bioorg. Med. Chem. Lett..

[4] Tohnishi M., Nakao H., Kohno E., Nishida T., Furuya T., Shimizu T., Seo A., Sakata K., Fujioka S. (2003). Phthalic Acid Diamide Derivatives, Fluorine-Containing Aniline Compounds as Starting Material, Agricultural and Horticultural Insecticides, and A Method for Application of the Insecticides. U.S. Patent.

[5] Masali T., Yasokawa N., Tohnishi M., Nishimatsu T., Tsubata K., Inoue K., Motoba K., Hirooka T. (2006). Flubendiamide, a novel Ca^2+^ channel modulator, reveals evidence for functional cooperation between Ca^2+^ pumps and Ca^2+^ release. Mol. Pharmacol..

[6] Lahm G.P., Cordova D., Barry J.D. (2009). New and selective ryanodine receptor activators for insect control. Bioorg. Med. Chem..

[7] Hughes K.A., Lahm G.P., Selby T.P., Stevenson T.M. (2004). Cyano Anthranilamide Insecticides.

[8] Akihiko Y., Yukiyishi W., Katsuaki W., Tetsuya M., Katsuhiko S., Eiichi S., Akira E. (2007). Insecticidal 3-Acylaminobenzanilides.

[9] Pierre J., Patricia D., William L., Peter M., Thomas P., Peter R., Werner Z. (2007). Insecticidal Compounds.

[10] Zhang Y.B. (2012). The insecticedes of ryanodine receptor inhibitors and varieties structural character and mechanism. Agrochemicals.

[11] Li Y.X., Mao M.Z., Li Y.M., Xiong L.X., Li Z.M., Xu J.Y. (2011). Modulations of high-voltage activated Ca^2+^channels in the central neurones of *Spodoptera exigua* by chlorantraniliprole. Physiol. Entomol..

[12] Cordova D., Benner E.A., Sacher M.D., Rauh J.J., Sopa J.S., Lahm G.P., Selby T.P., Stevenson T.M., Flexner L., Gutteridge S. (2006). Anthranilicdiamides: A new class of insecticides with a novel mode of action, ryanodine receptor activation. Pestic. Biochem. Physiol..

[13] Wing K.D. (1988). RH-5849, a nonsteroidal ecdysone agonist: effects on a drosophila cell line. Science.

[14] Sun R.F., Zhang Y.L., Chen L., Li Y.Q., Li Q.S., Song H.B., Huang R.Q., Bi F.C., Wang Q.M. (2009). Design, synthesis, and insecticidal activities of new *N*-benzoyl-*N*'-phenyl-*N*'-sulfenylureas. J. Agric. Food Chem..

[15] Wang H., Yang Z.K., Fan Z.J., Wu Q.J., Zhang Y.J., Mi N., Wang S.X., Zhang Z.C., Song H.B., Liu F. (2011). Synthesis and insecticidal activity of *N*-tert-Butyl-*N*,*N*'-diacylhydrazines containing 1,2,3-Thiadiazoles. J. Agric. Food Chem..

[16] Yanagi M., Tsukamoto Y., Watanabe T., Kawagishi A. (2006). Development of a novel lepidopteran insect control agent chromafenozide. J. Pestic. Sci..

[17] Aguirre O.U., Martínez A.M., Campos-García J., Hernández L., Figueroa J.I., Lobit P., Viñuela E., Chavarrieta J.M., Smagghe G., Pineda1 S. (2013). Foliar persistence and residual activity of methoxyfenozide against beet armyworm (Lepidoptera: Noctuidae). Insect Sci..

[18] Salgado V.L., Hayashi J.H. (2007). Metaflumizone is a novel sodium channel blocker insecticide. Vet. Parasitol..

[19] Hempel K., Hess F.G., Bogi C., Fabian E., Hellwig J., Fegert I. (2007). Toxicological properties of metaflumizone. Vet. Parasitol..

[20] Che Z.P., Zhang S.Y., Shao Y.H., Fan L.L., Xu H., Yu X., Zhi X.Y., Yao X.J., Zhang R. (2013). Synthesis and quantitative structure-activity relationship (QSAR) study of novel *N*-arylsulfonyl-3-acylindole arylcarbonyl hydrazone derivatives as nematicidal agents. J. Agric. Food Chem..

[21] Qu H., Yu Y., Zhi X.Y., Lv M., Xu H. (2013). Natural-product-based insecticidal agents 14.Semisynthesis and insecticidal activity of new piperine-based hydrazone derivatives against *Mythimna separata* Walker *in vivo*. Bioorg. Med. Chem. Lett..

[22] Cao G., Han Z. (2006). Tebufenozide resistance selected in *Plutella xylostella* and its cross-resistance and fitness cost. Pest. Manag. Sci..

[23] Mao C.H., Wang Q.M., Huang R.Q., Bi F.C., Chen L., Liu Y.X., Shang J. (2004). Synthesis and insecticidal evaluation of novel *N*-oxalyl derivatives of tebufenozide. J. Agric. Food Chem..

[24] Zhang J.W., Hu Z., Li S.K., Wu W.J. (2011). Synthesis and insecticidal activities of new ester-derivatives of Celangulin-V. Int. J. Mol. Sci..

