# Supplementary material for: Design, Synthesis, and Insecticidal Activity of Some Novel Diacylhydrazine and Acylhydrazone Derivatives
Source: Molecules. 2015 Mar 30;20(4):5625–37. doi: 10.3390/molecules20045625 (PMC6272438; doi:10.3390/molecules20045625)
Supplement: Supplementary file 1 [file molecules-20-05625-s001.pdf]

Supplementary Materials

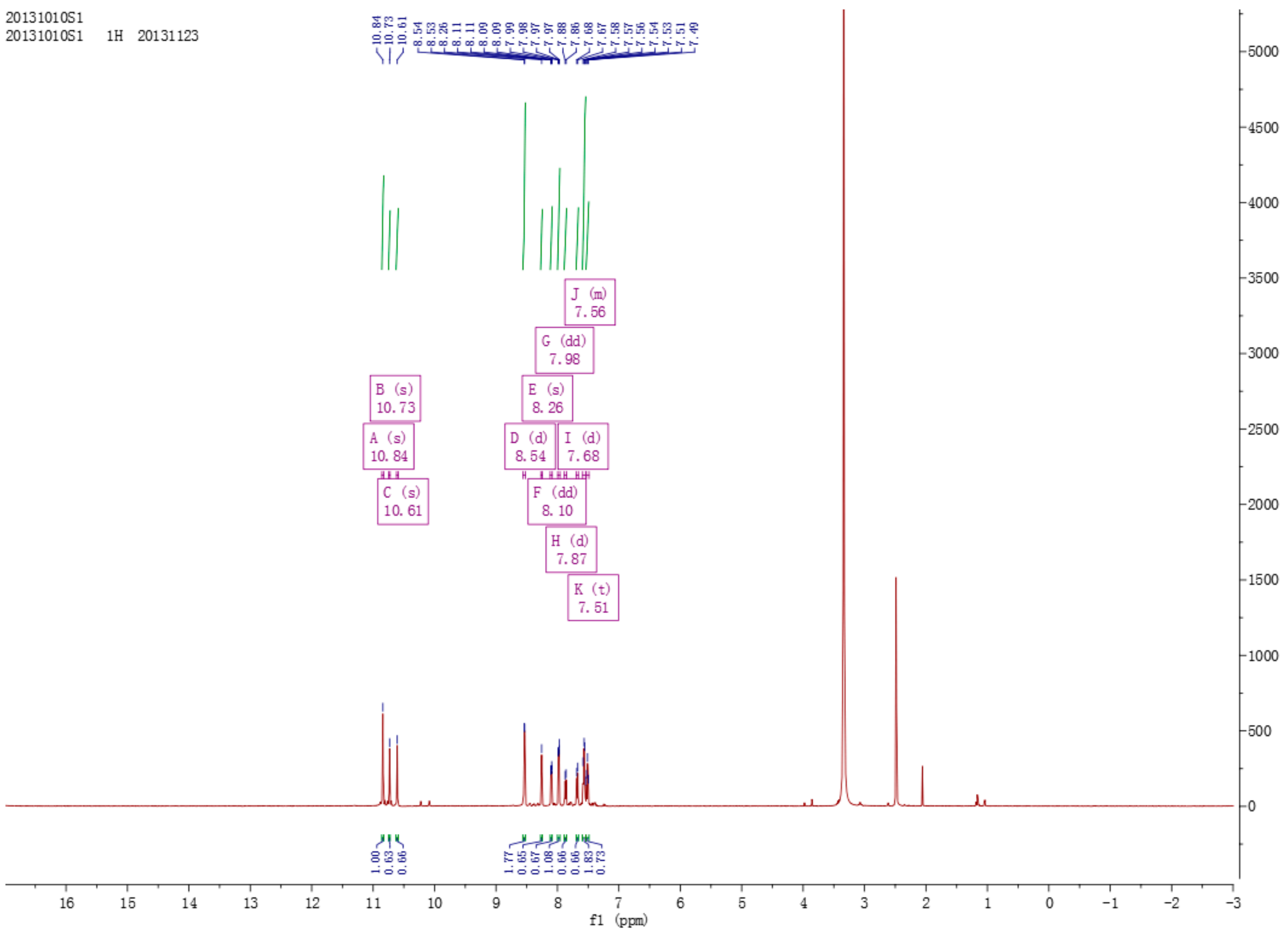

Figure S1. ^{1}H-NMR of compound **3a**.

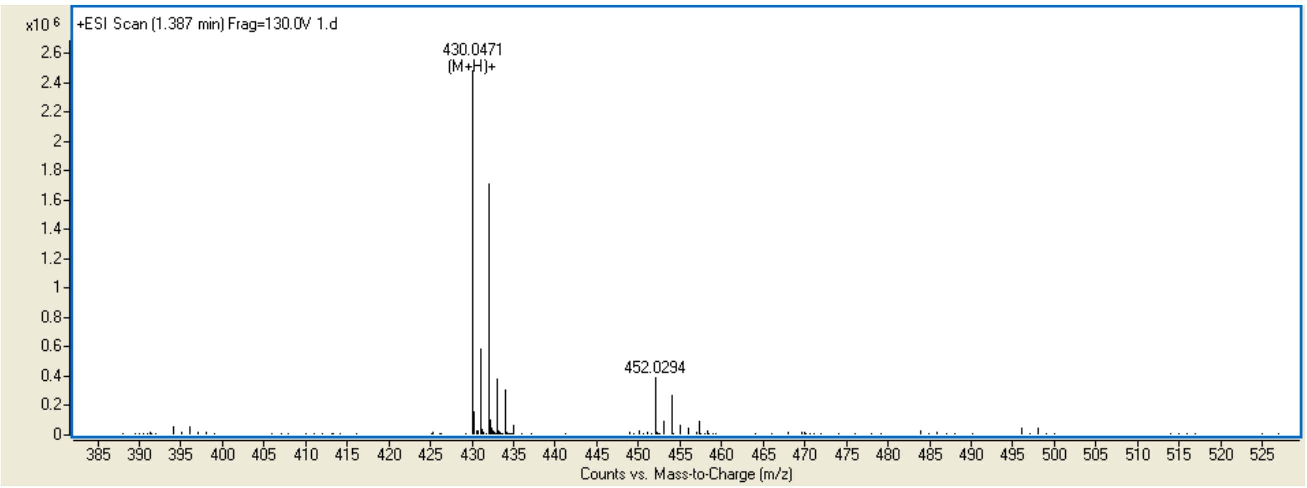

Figure S2. HR-ESI-MS of compound **3a**.

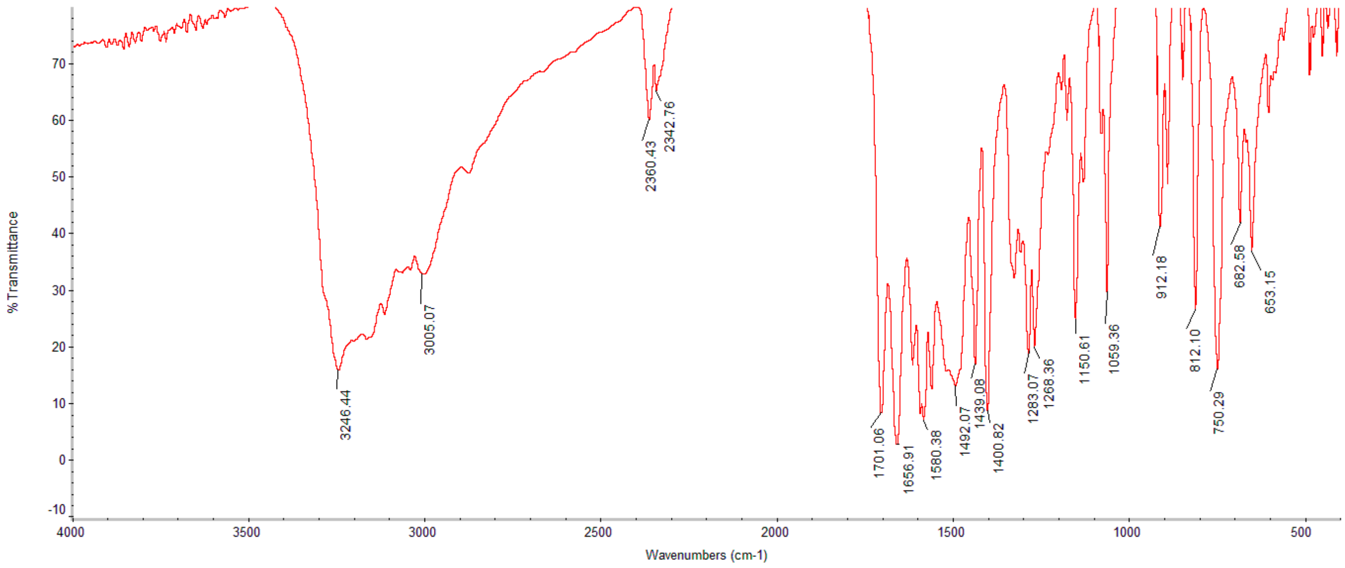

Figure S3. IR of compound **3a**.

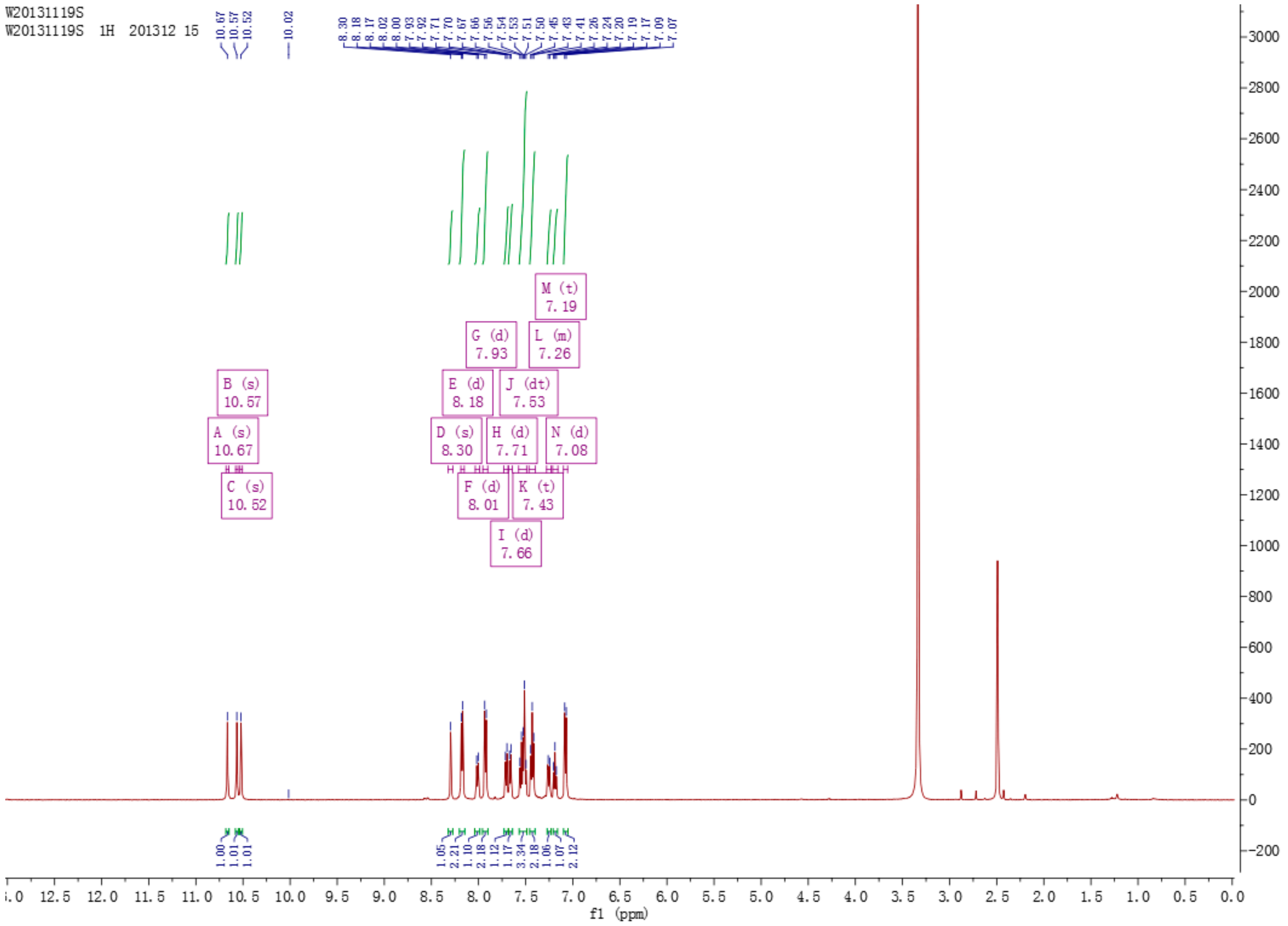

Figure S4. ^{1}H-NMR of compound **3b**.

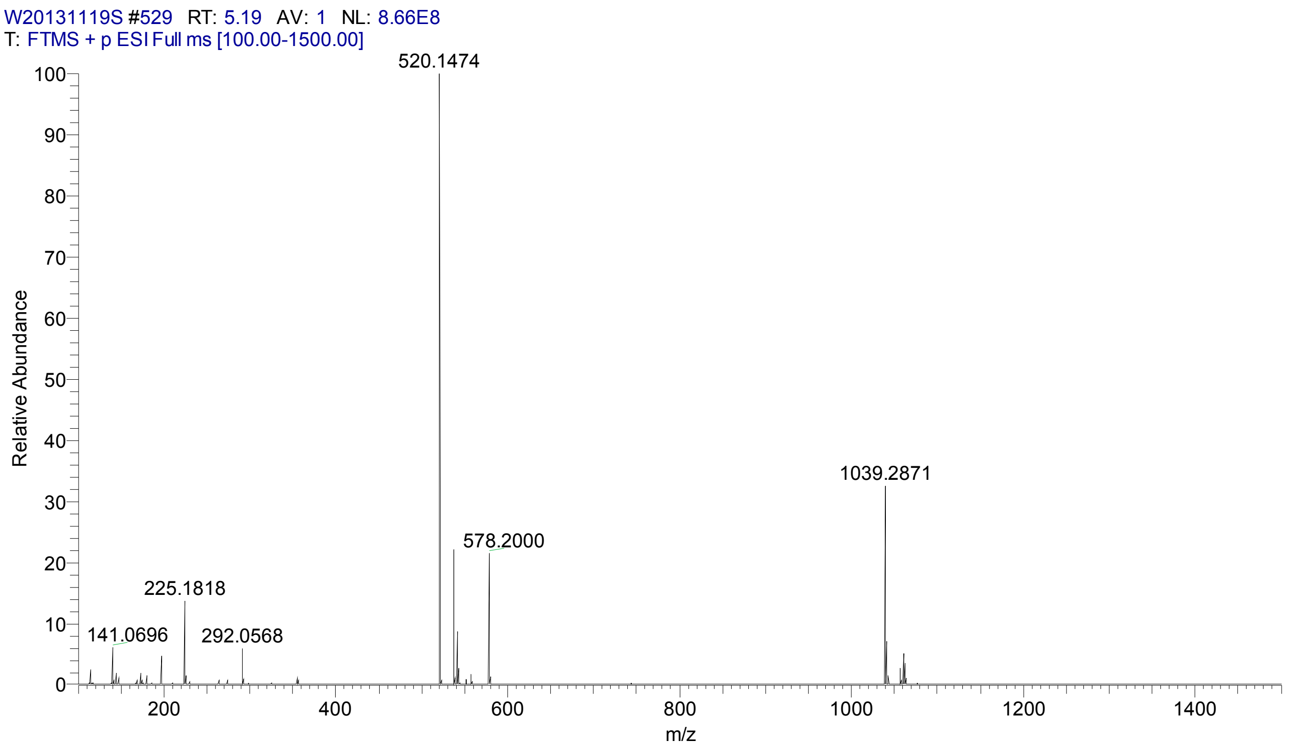

Figure S5. HR-ESI-MS of compound **3b**.

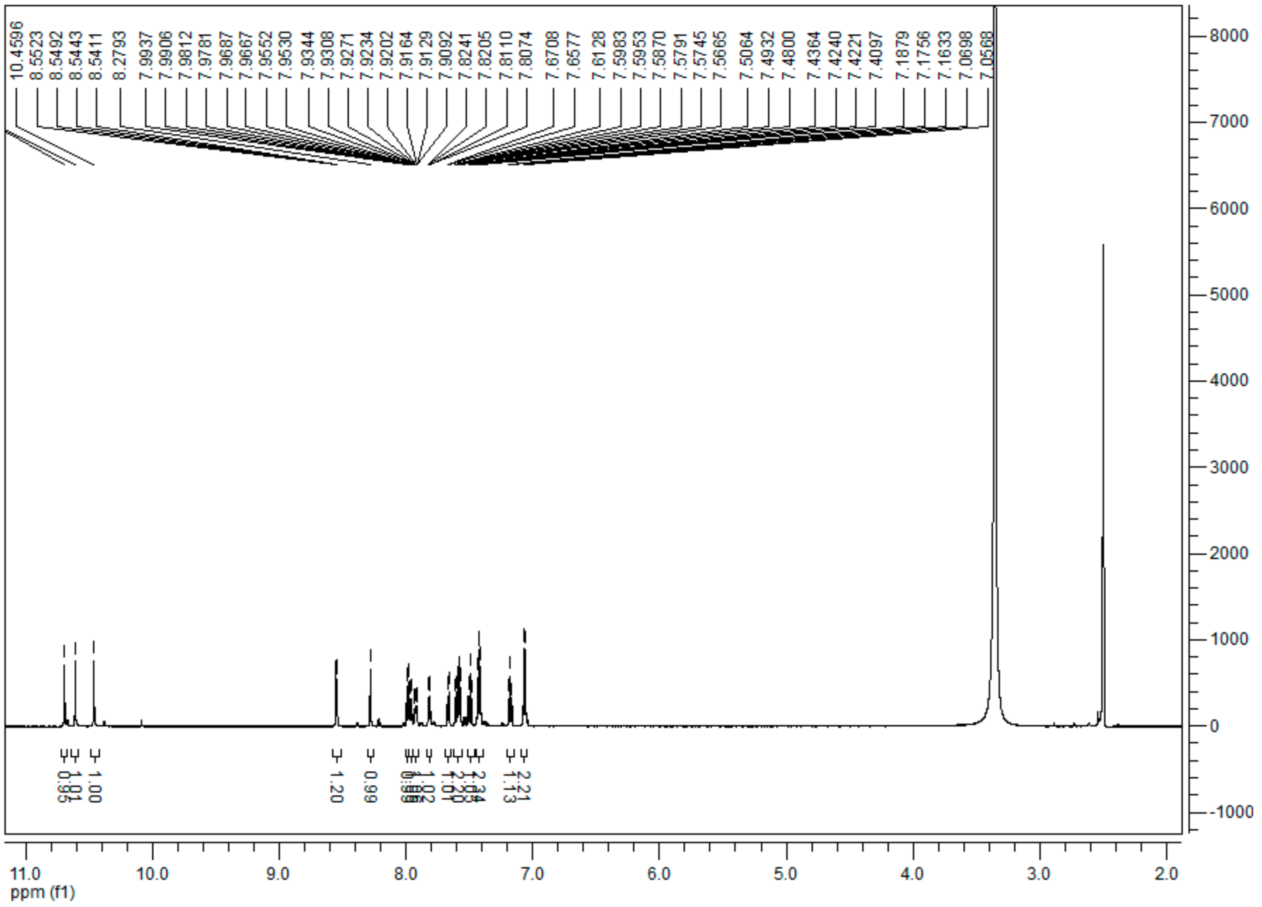

Figure S6. ^{1}H-NMR of compound **3c**.

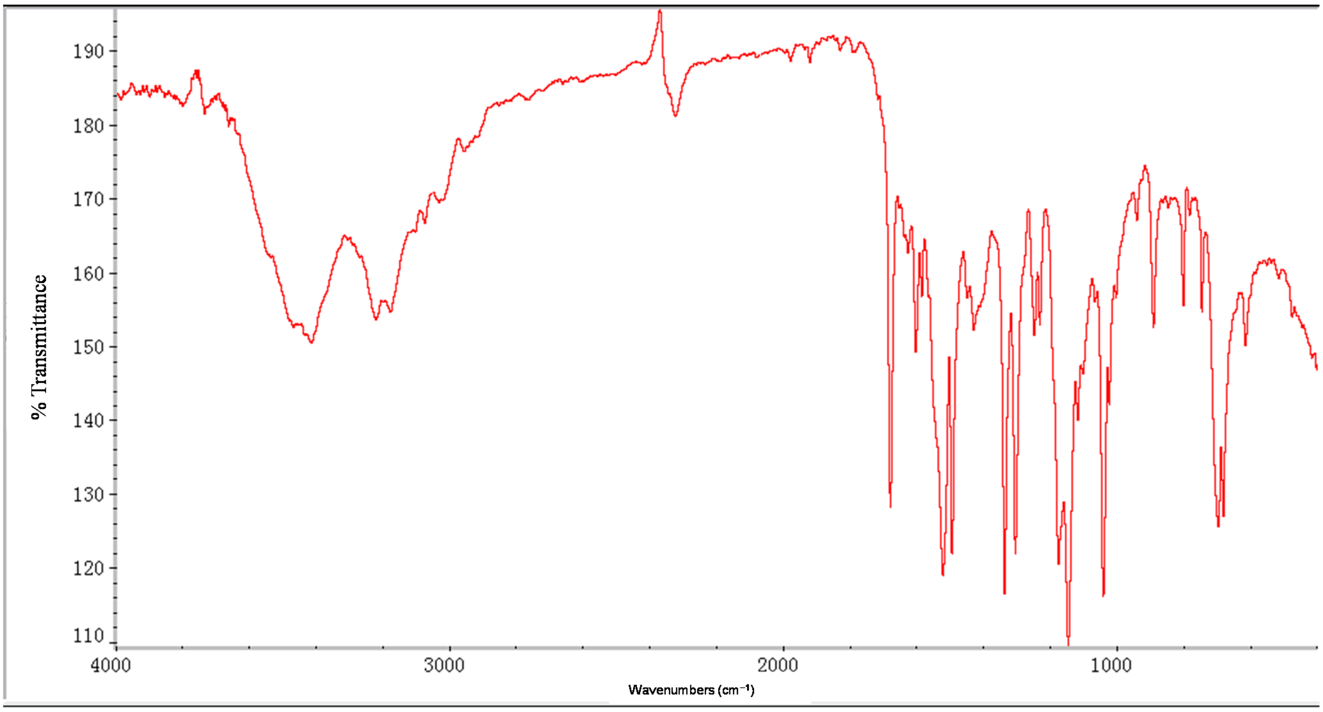

Figure S7. IR of compound **3c**.

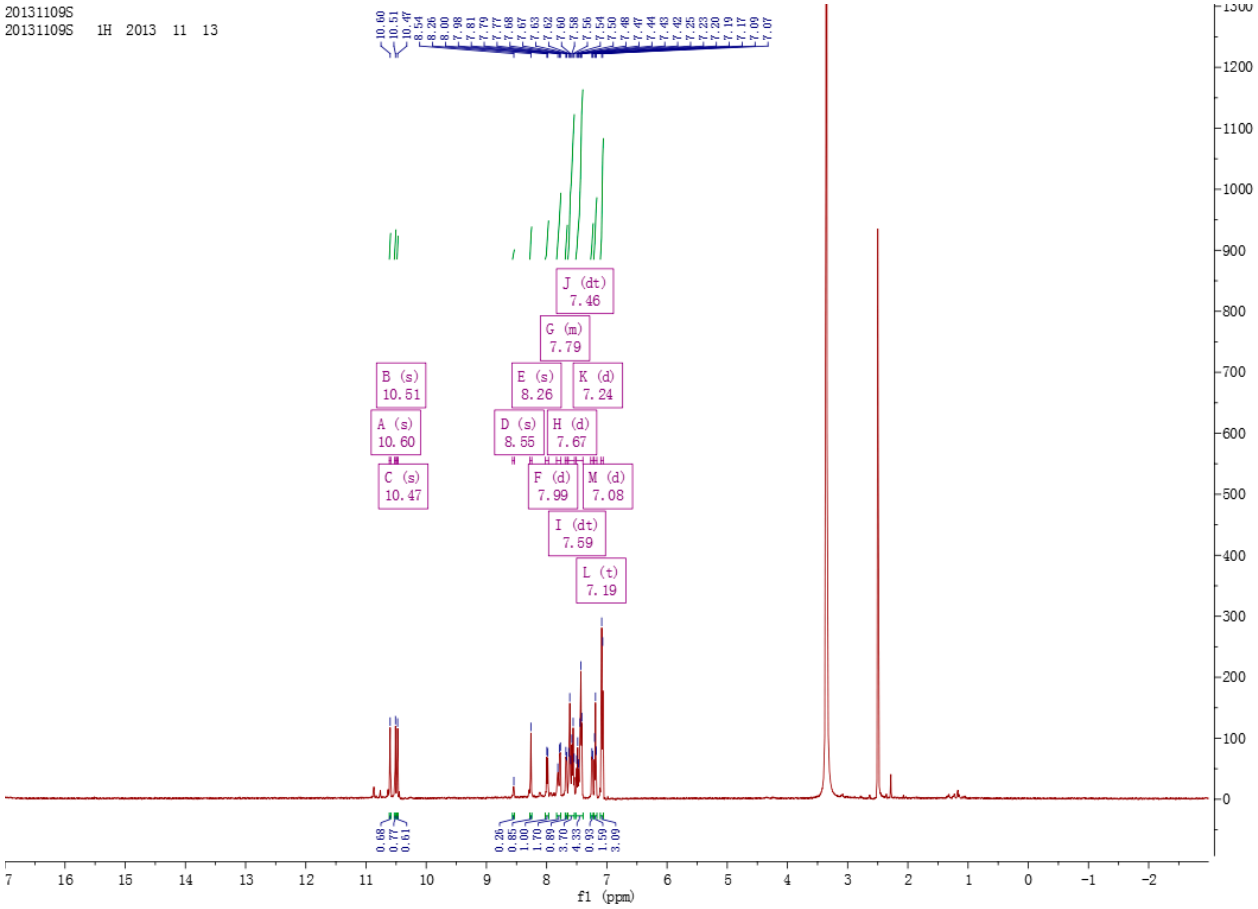

Figure S8. ^{1}H-NMR of compound **3d**.

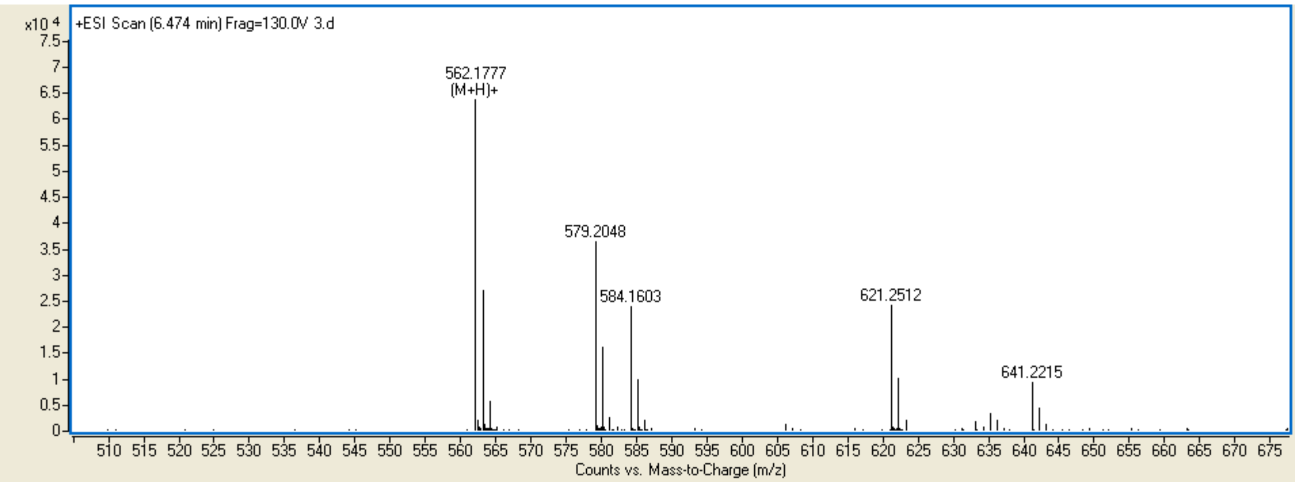

Figure S9. HR-ESI-MS of compound **3d**.

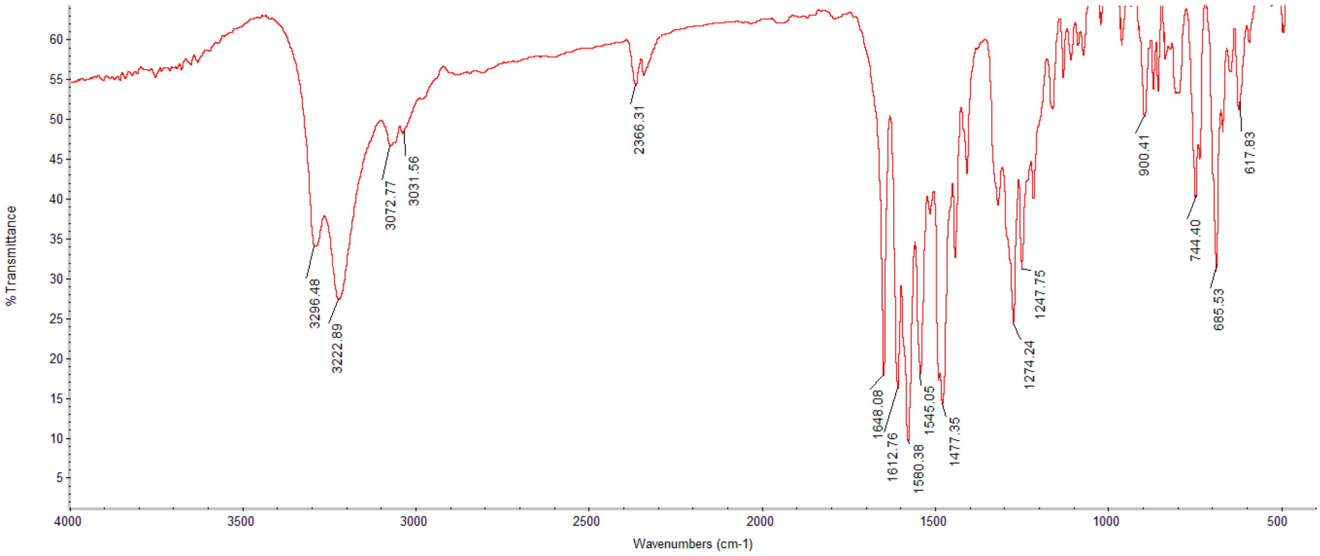

Figure S10. IR of compound **3d**.

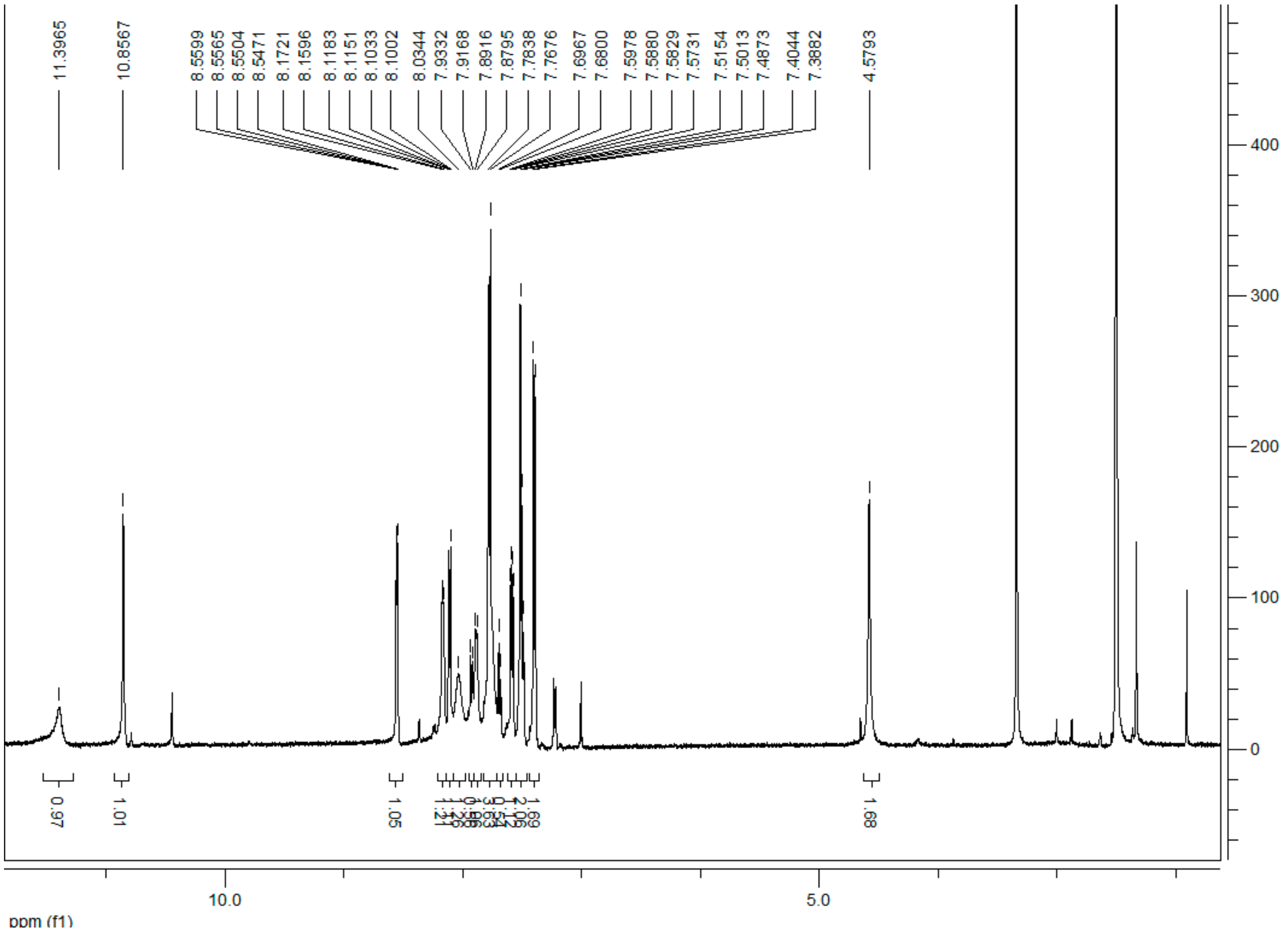

Figure S11. ^{1}H-NMR spectra of compound **4a**.

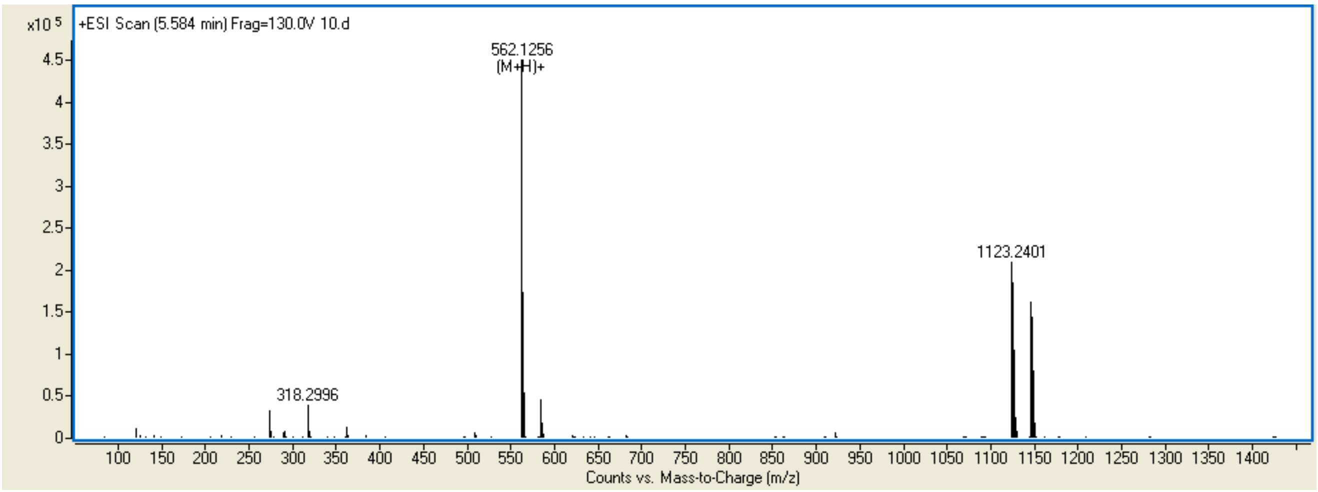

Figure S12. MS of compound **4a**.

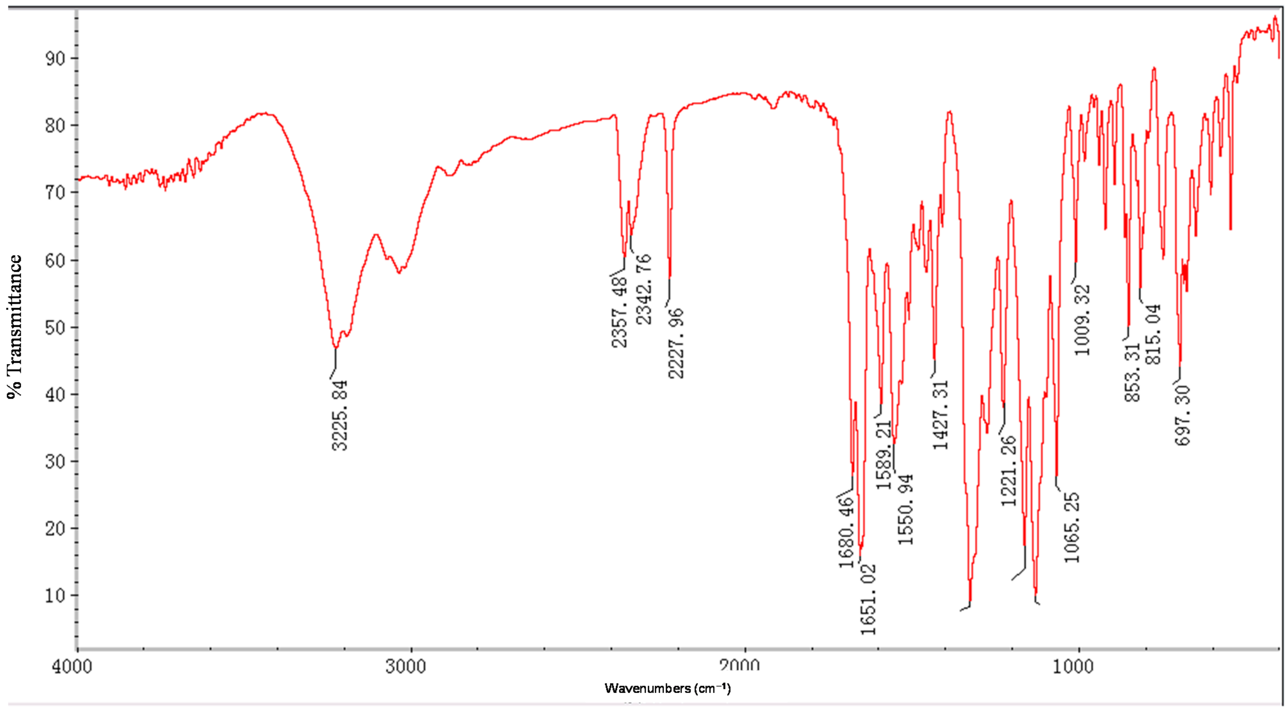

Figure S13. IR spectra of compound **4a**.

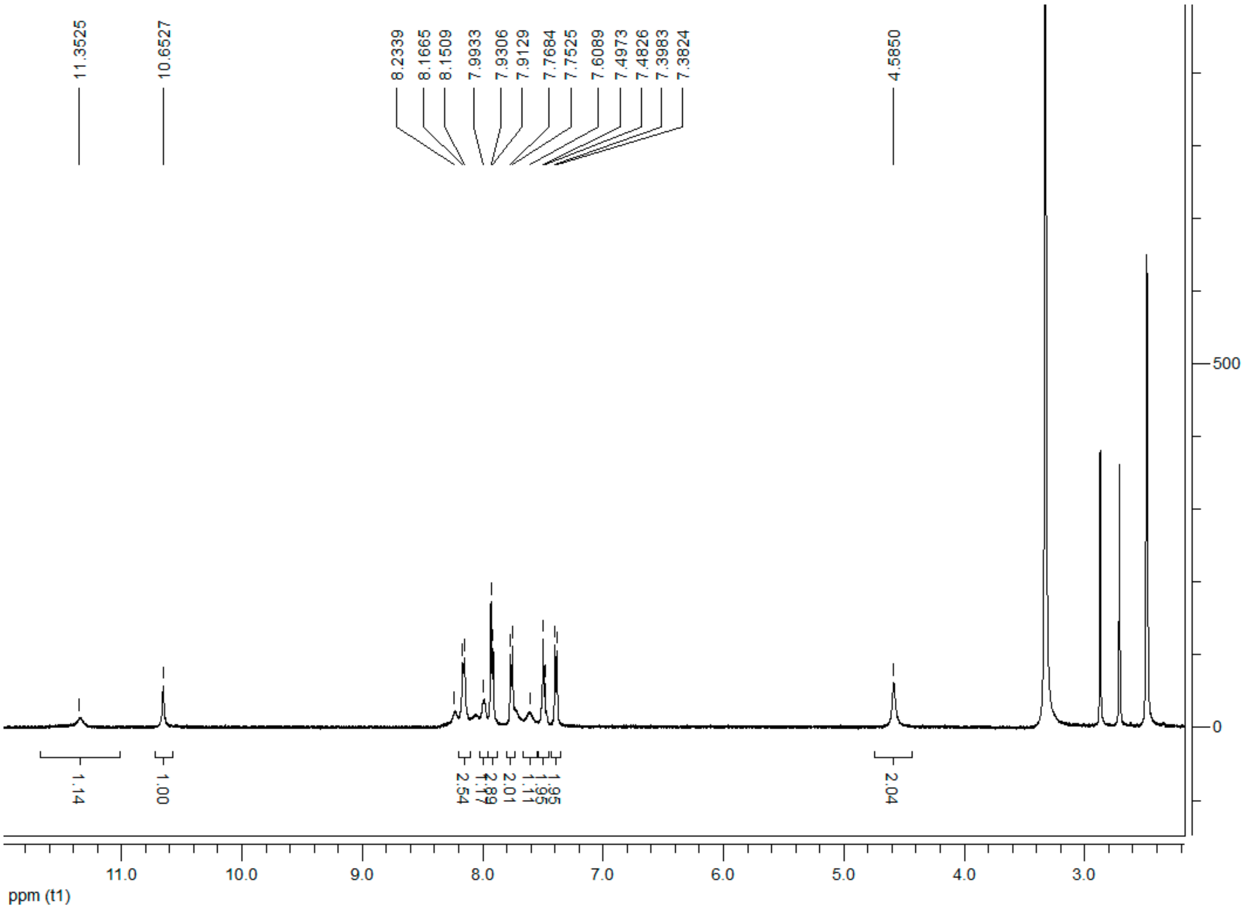

Figure S14. ^{1}H-NMR spectra of compound **4b**.

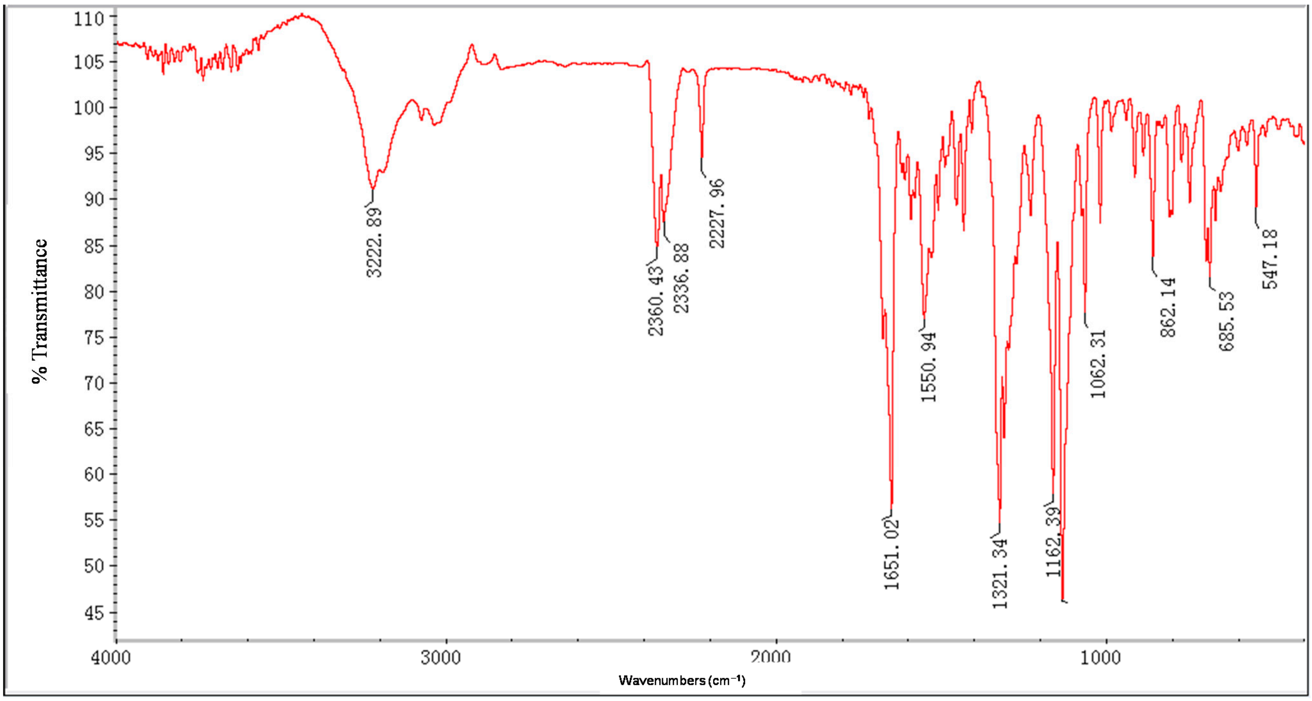

Figure S15. IR spectra of compound **4b**.

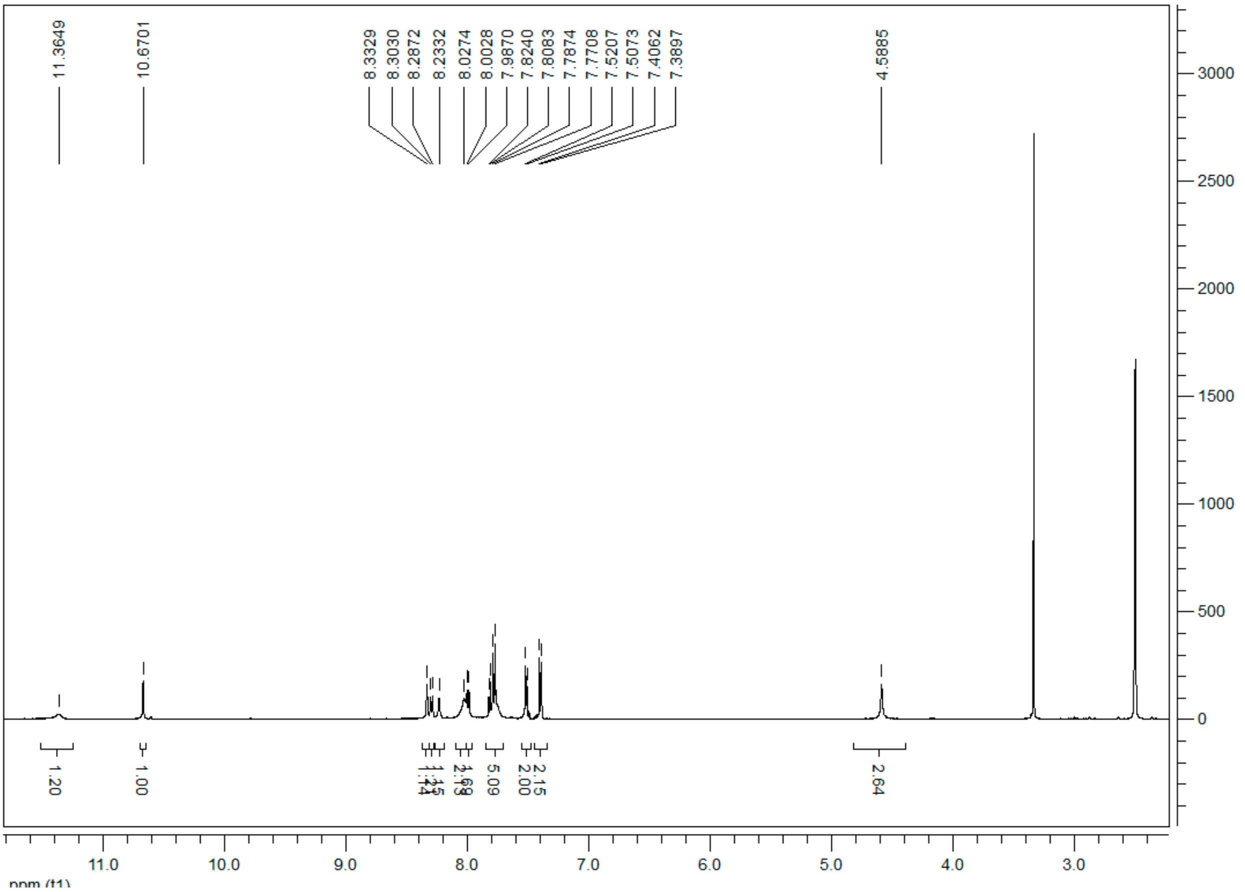

Figure S16. ^{1}H-NMR spectra of compound **4c**.

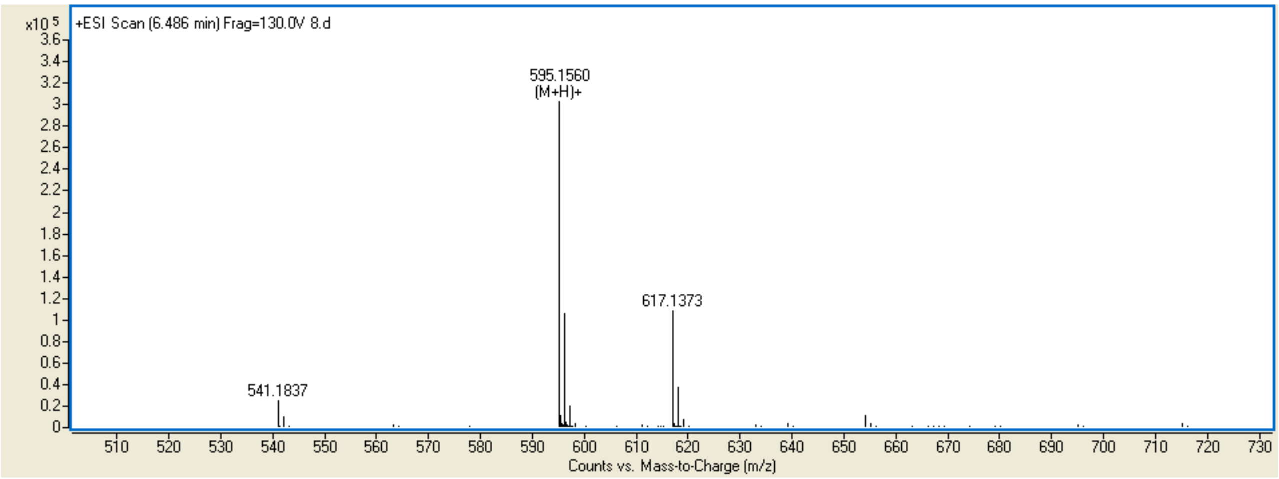

Figure S17. MS of compound **4c**.

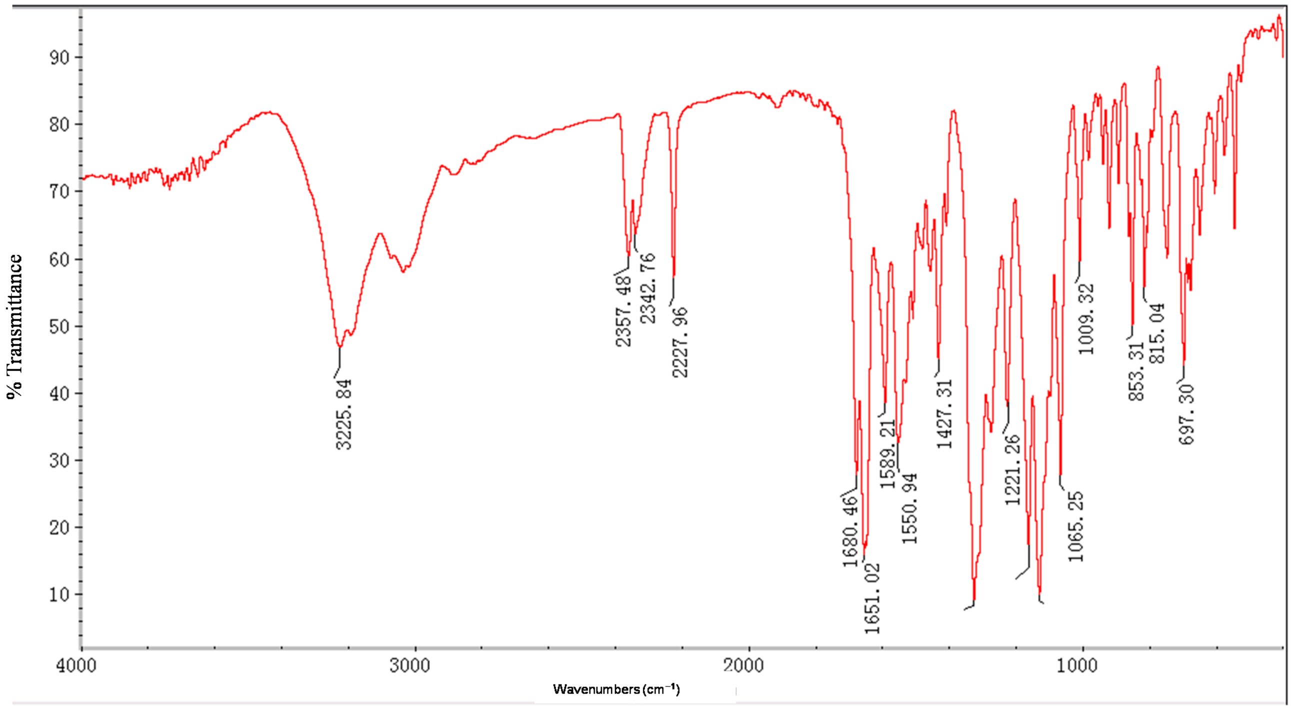

Figure S18. IR of compound **4c**.

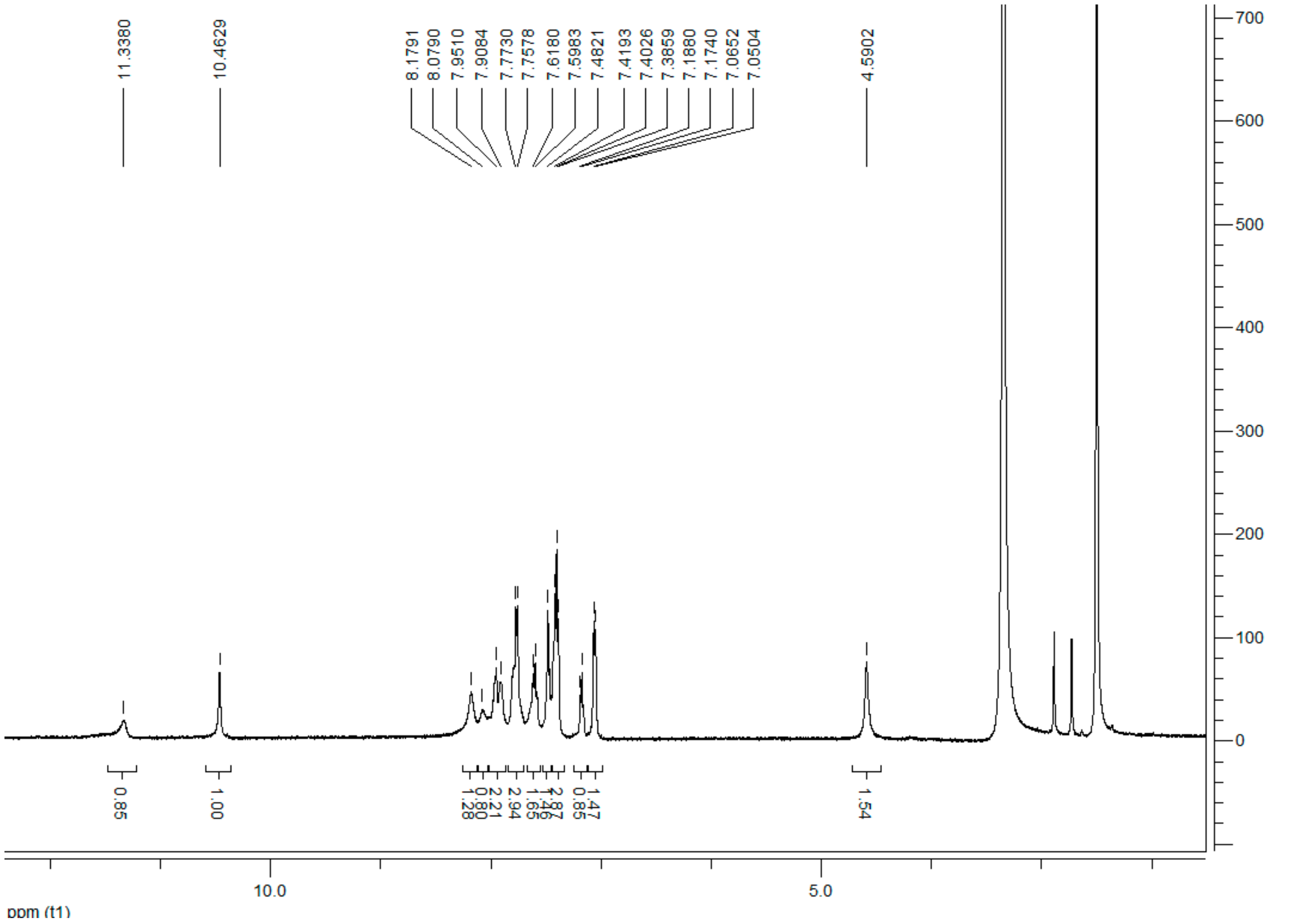

Figure S19. ^{1}H-NMR spectra of compound **4d**.

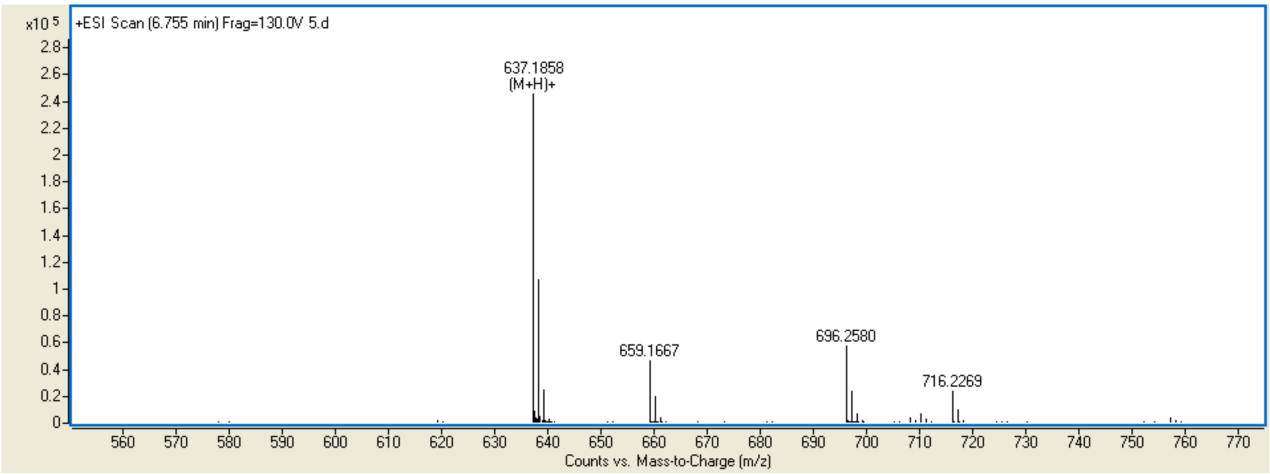

Figure S20. MS of compound **4d** W20131123F.

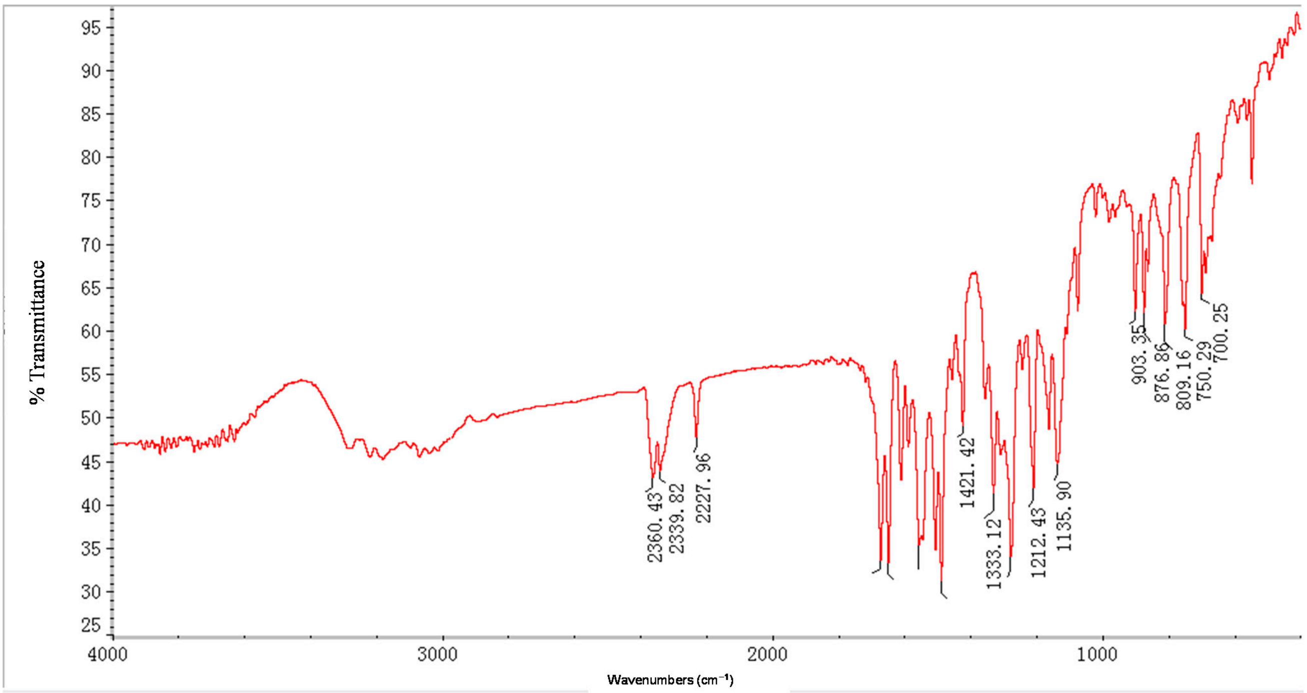

Figure S21. IR of compound **4d**.

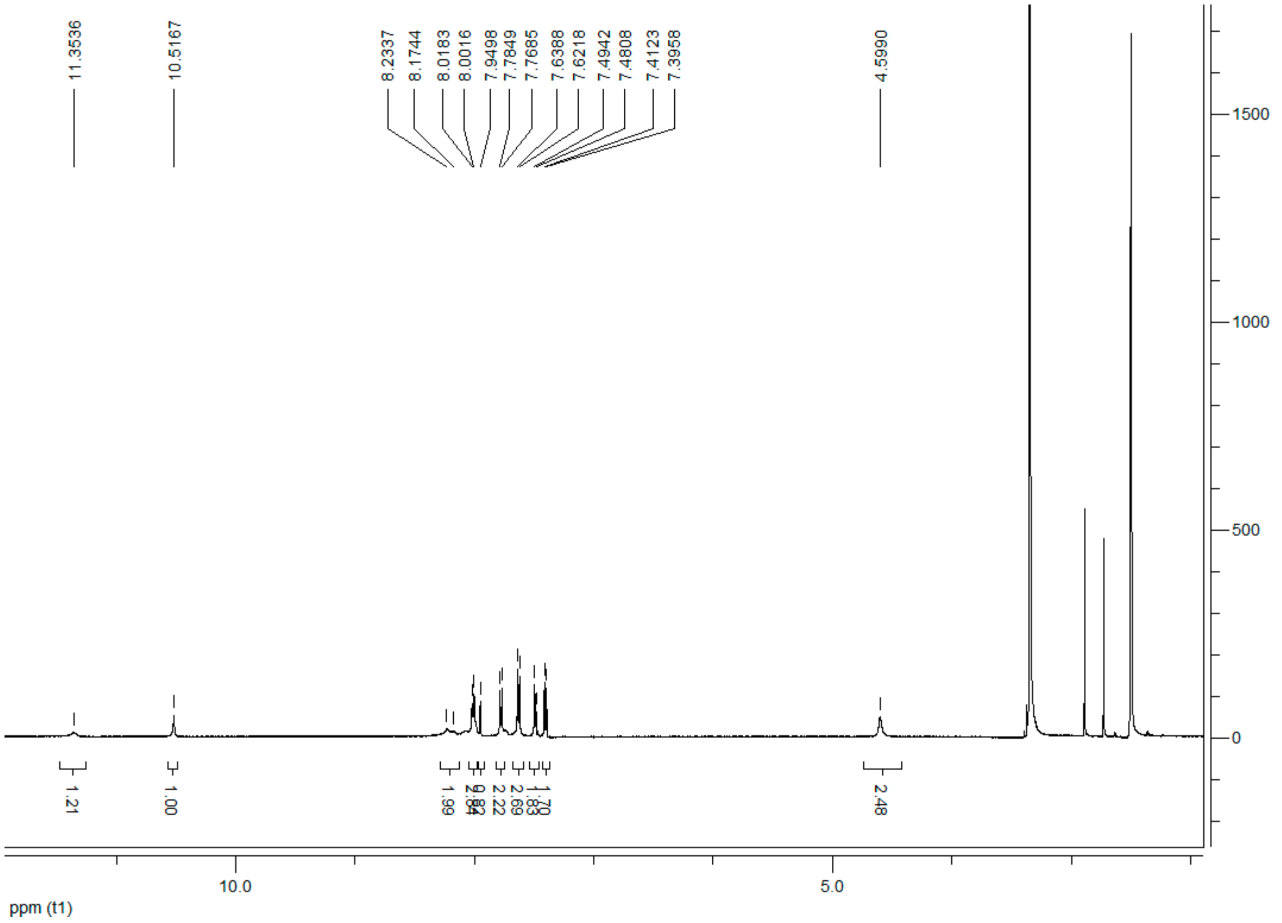

Figure S22. ^{1}H-NMR spectra of compound **4e**.

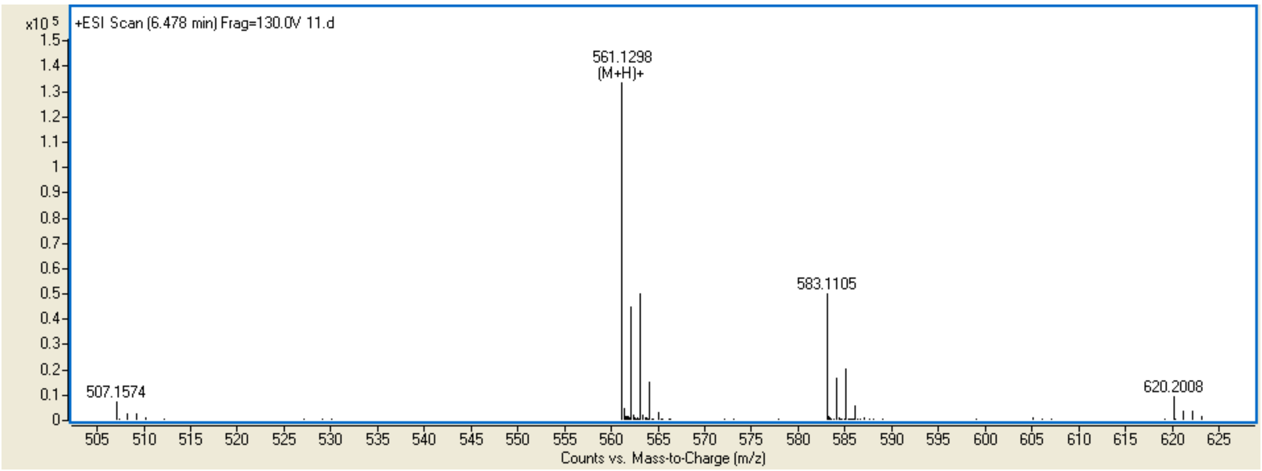

Figure S23. MS of compound **4e**.

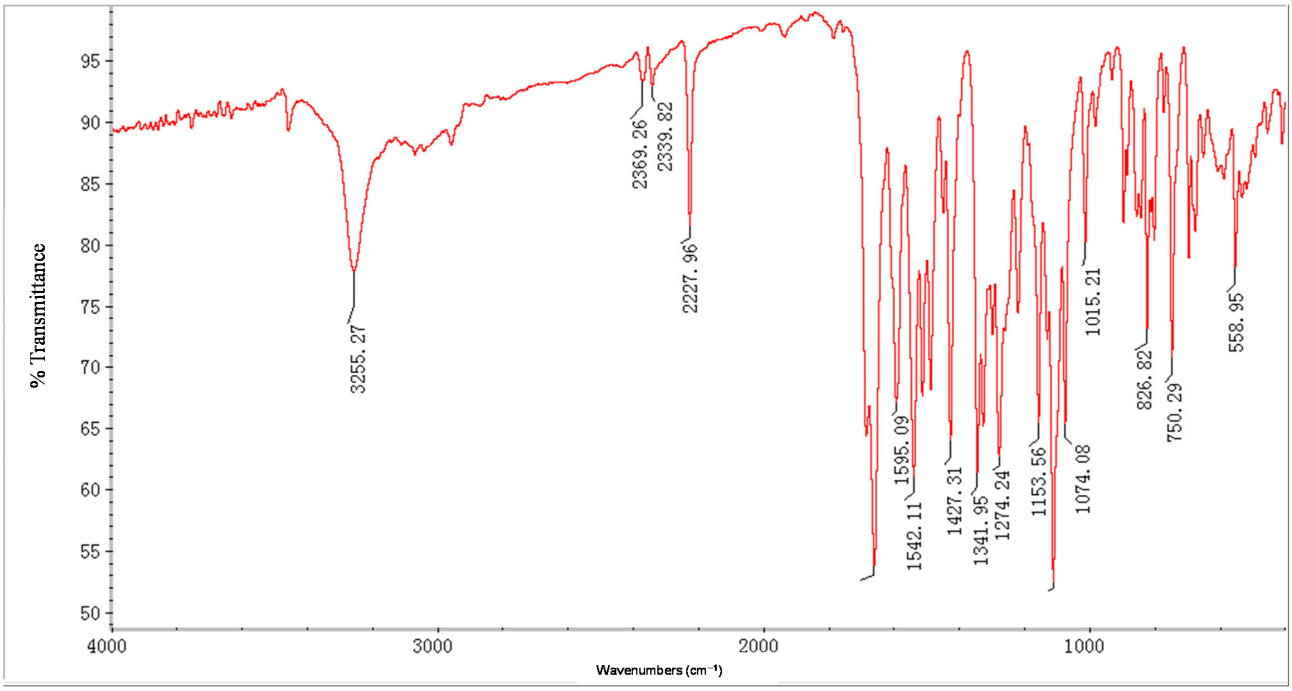

Figure S24. IR of compound **4e**.

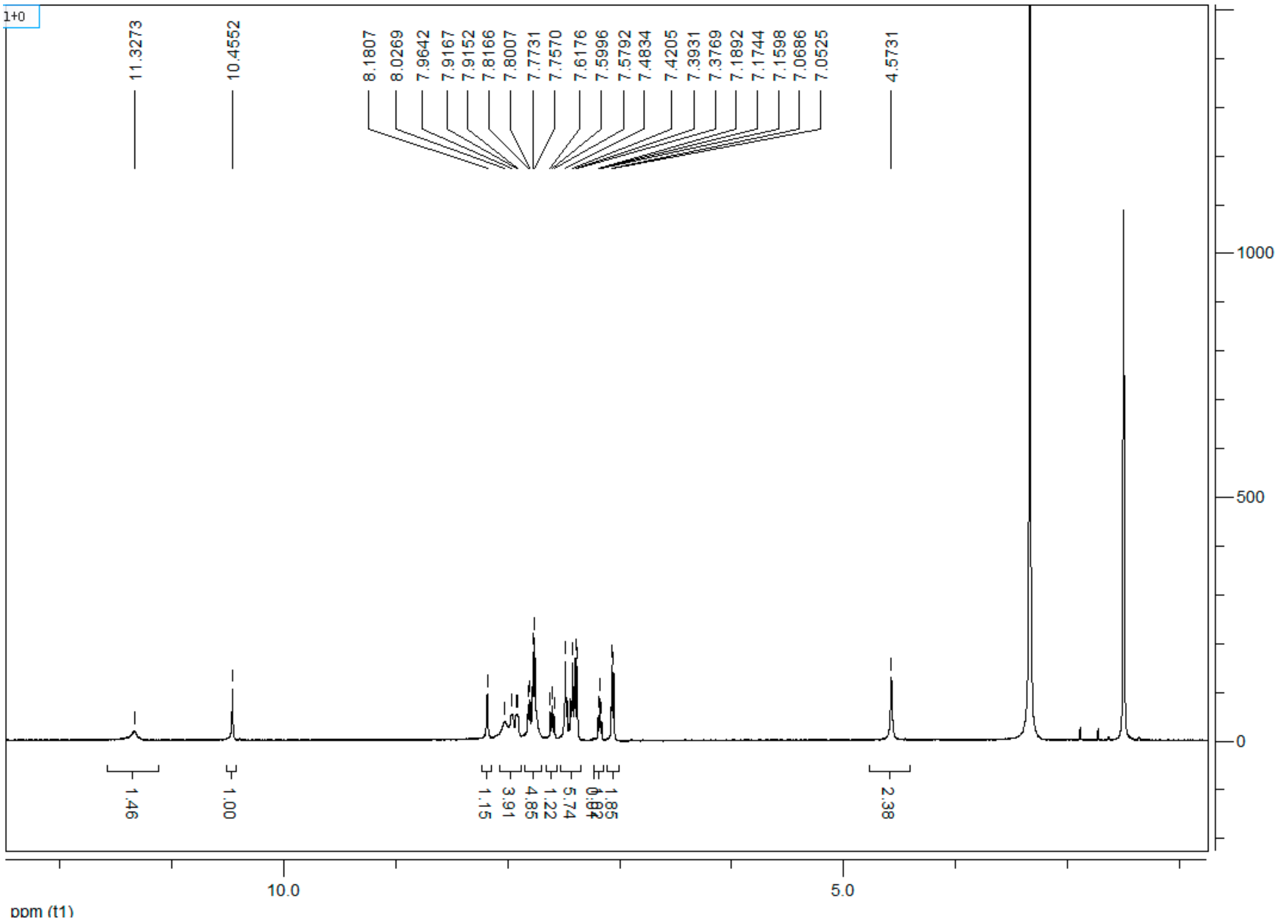

Figure S25. ^{1}H-NMR spectra of compound **4f**.

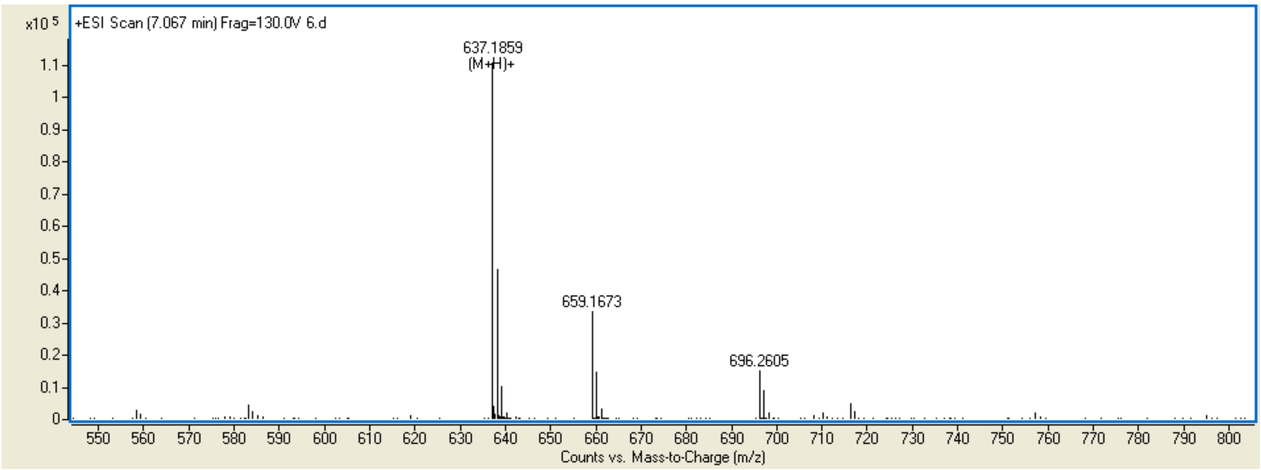

Figure S26. MS of compound **4f**.

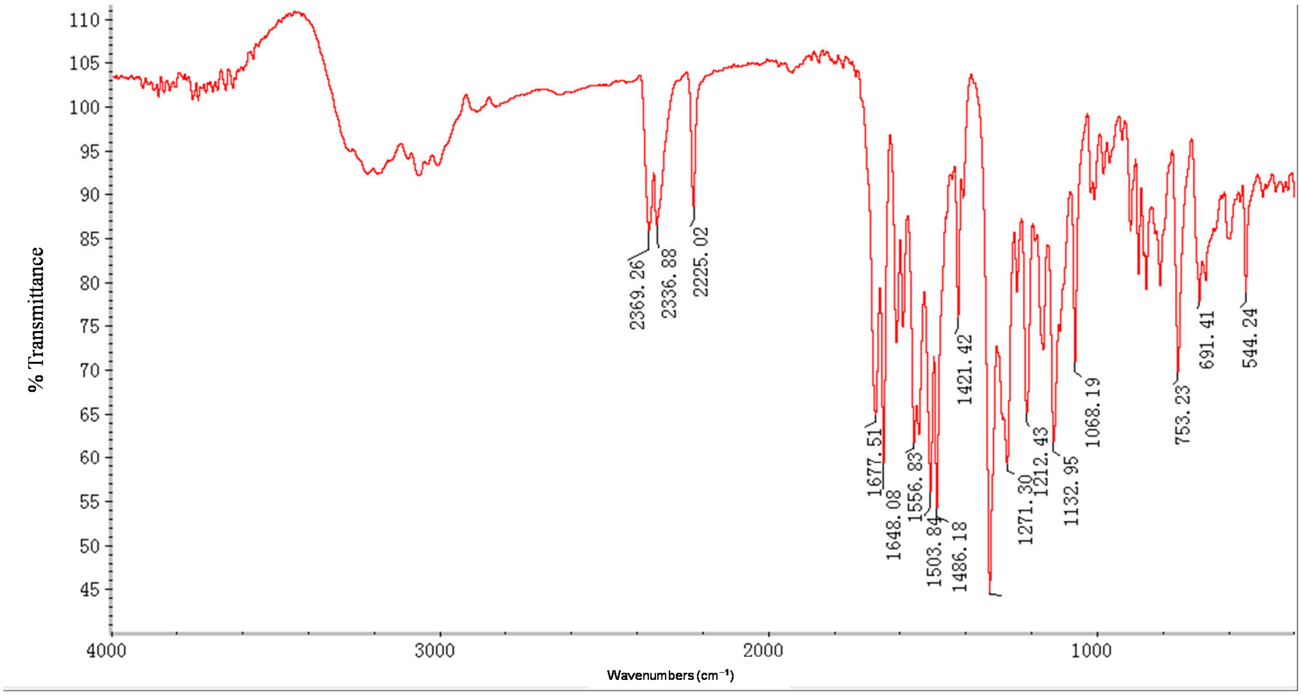

Figure S27. IR spectra of compound **4f**.

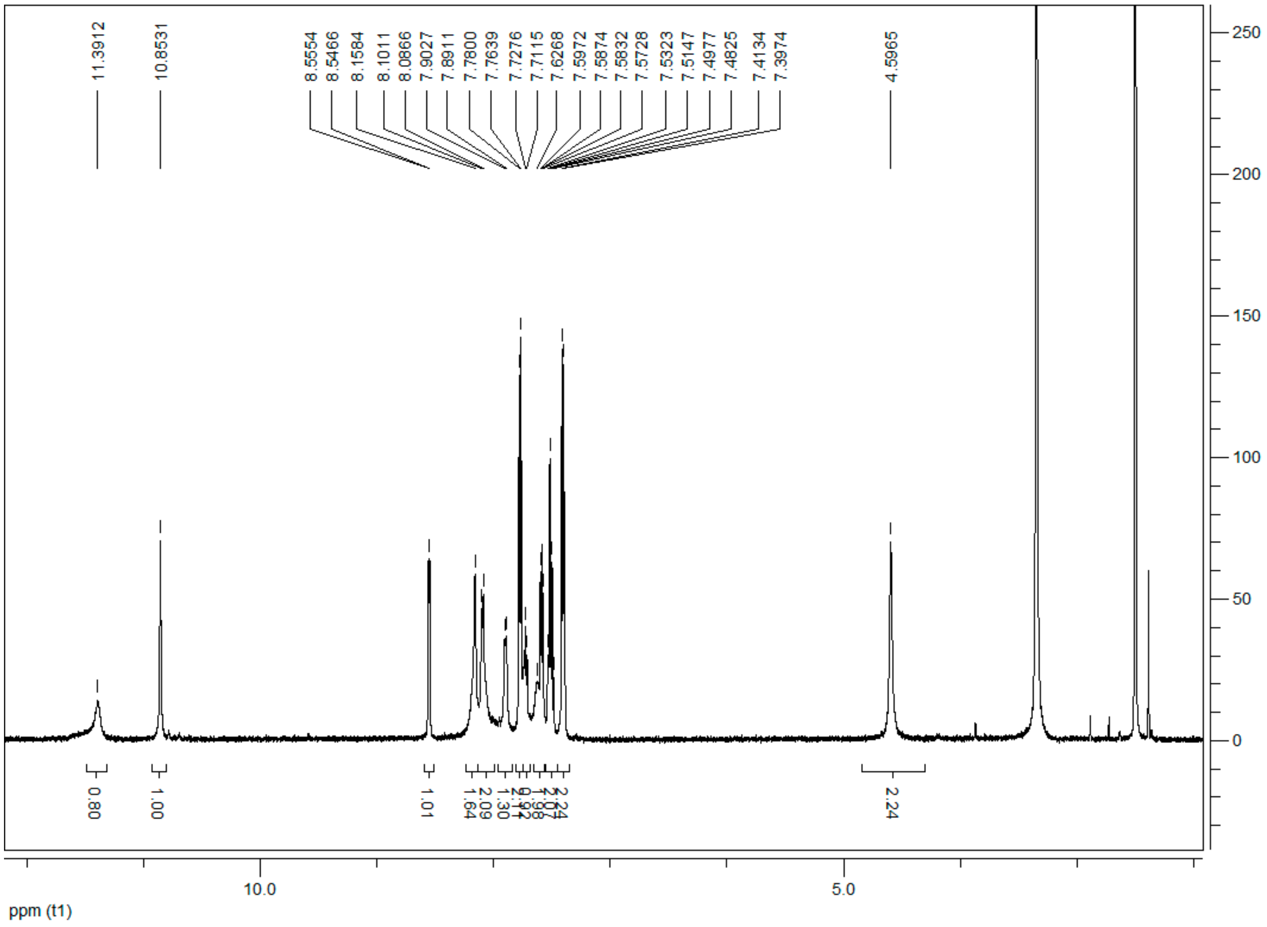

Figure S28. ^{1}H-NMR spectra of compound **4g**.

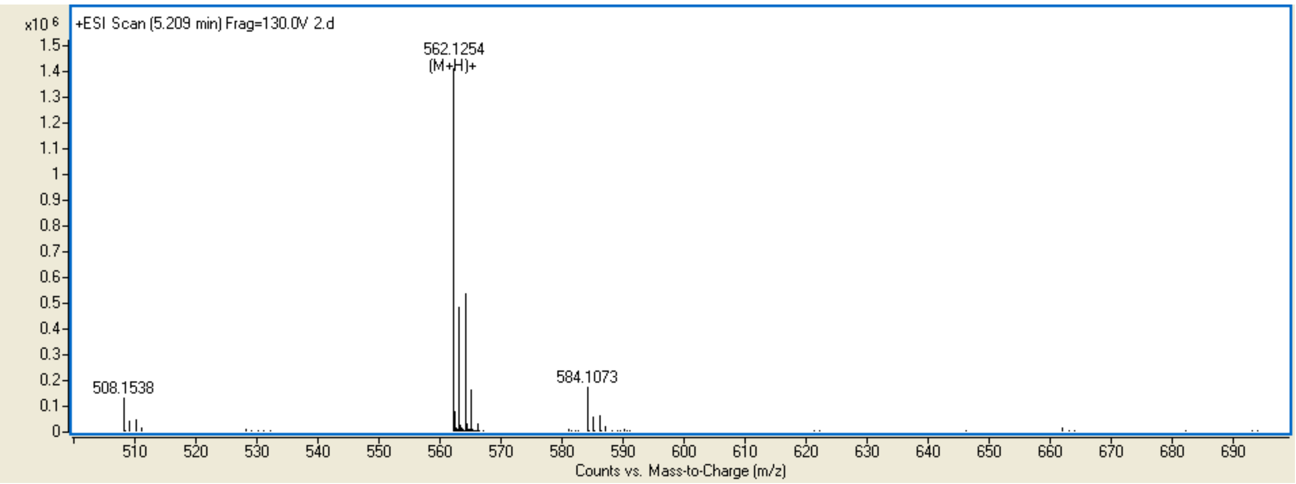

Figure S29. MS spectra of compound **4g**.

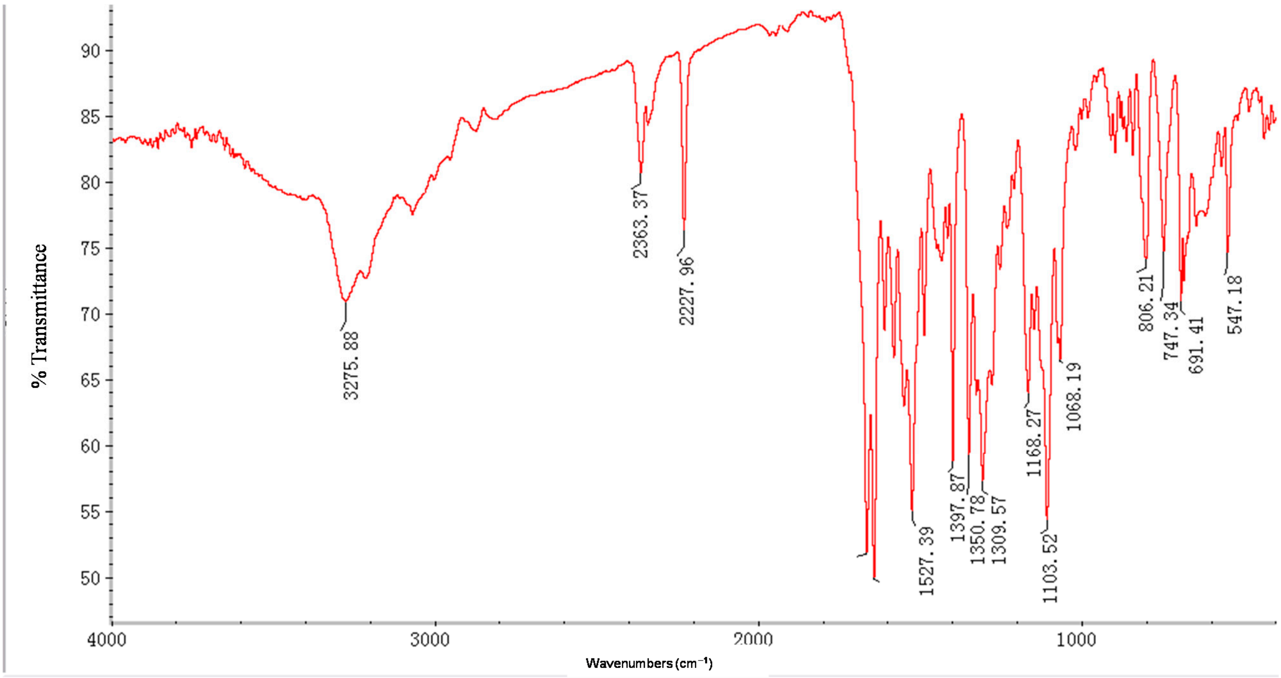

Figure S30. IR spectra of compound **4g**.

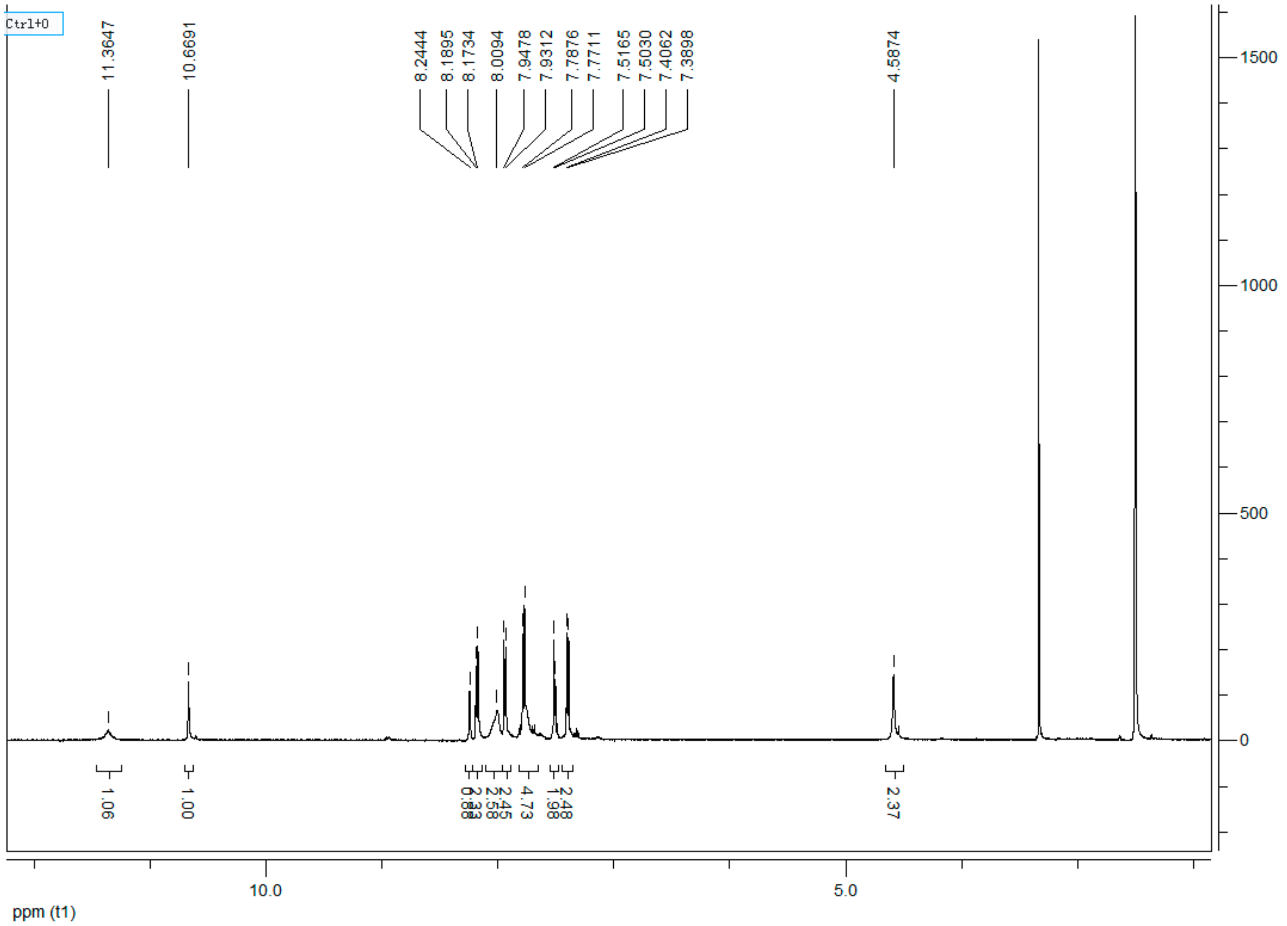

Figure S31. ^{1}H-NMR spectra of compound **4h**.

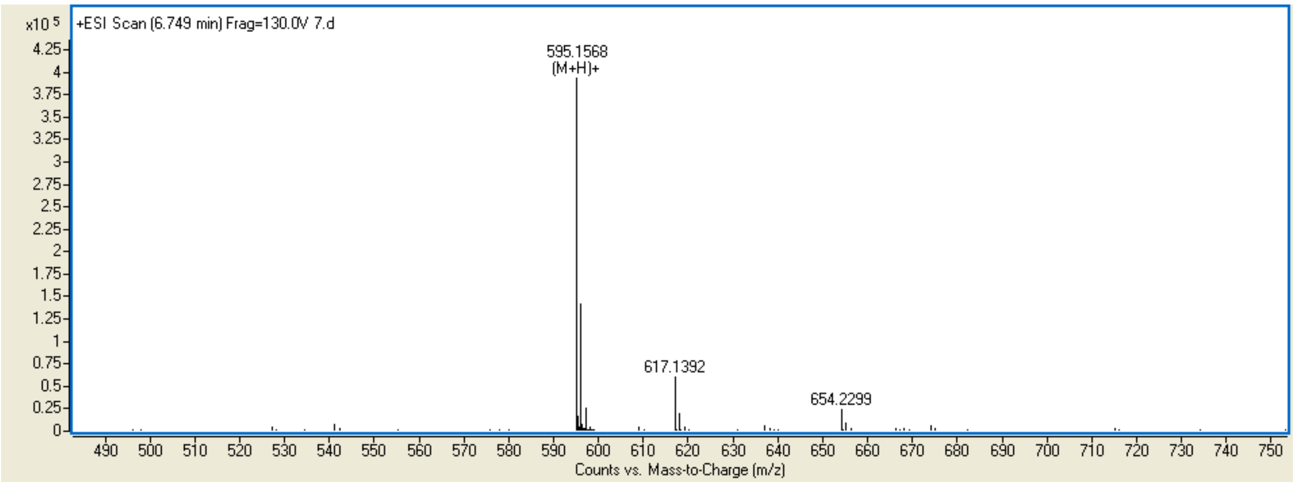

Figure S32. MS of compound **4h**.

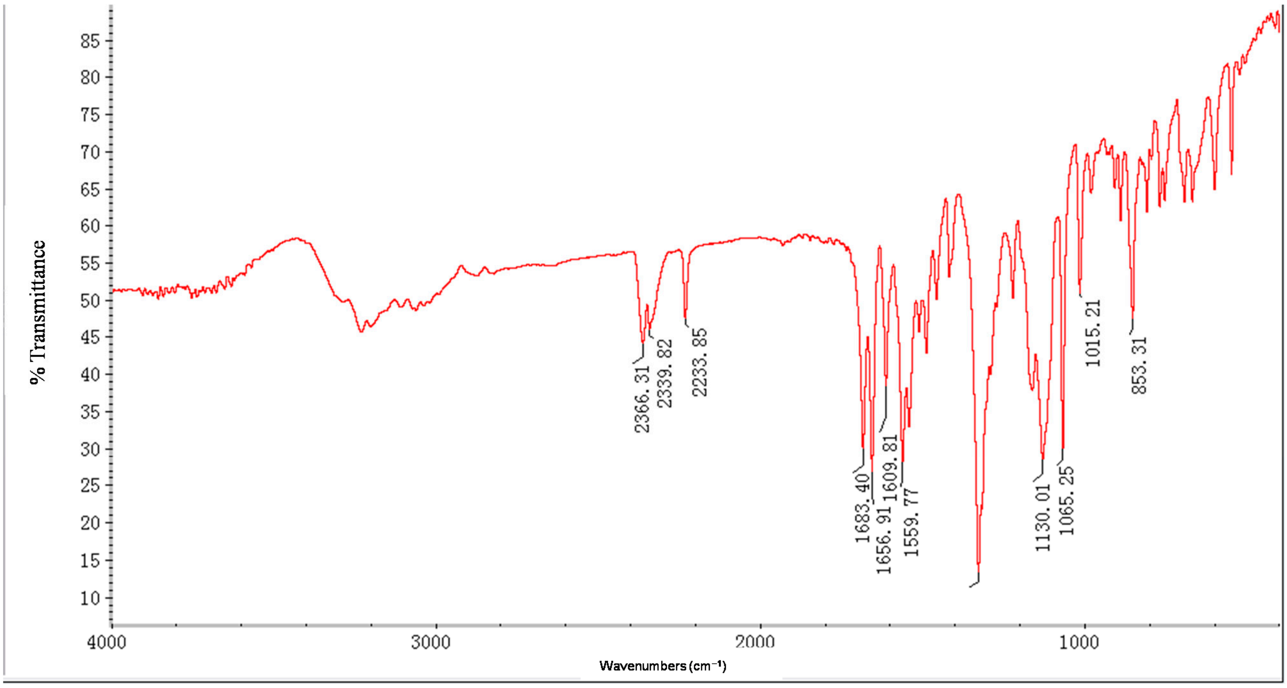

Figure S33. IR of compound **4h**.

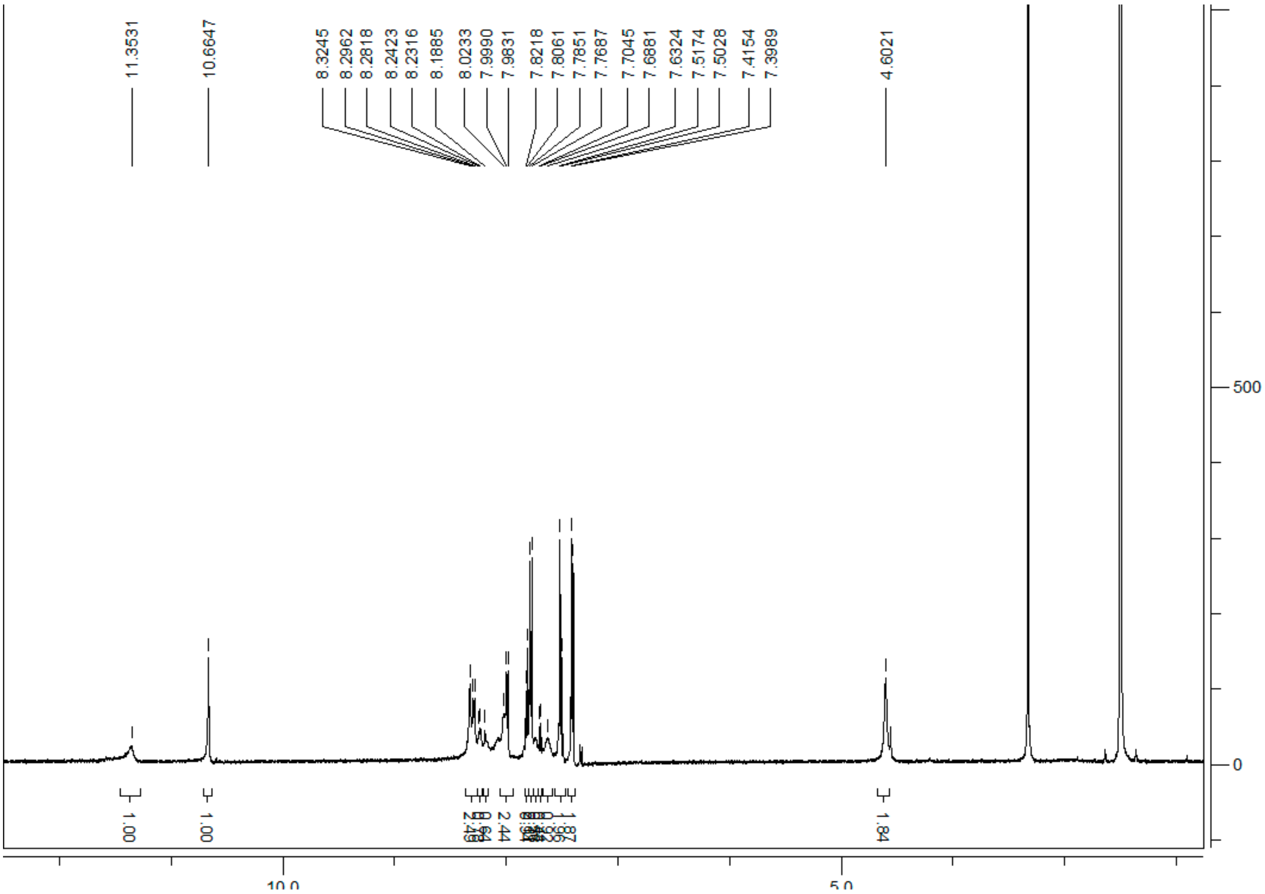

Figure S34. ^{1}H-NMR spectra of compound **4i**.

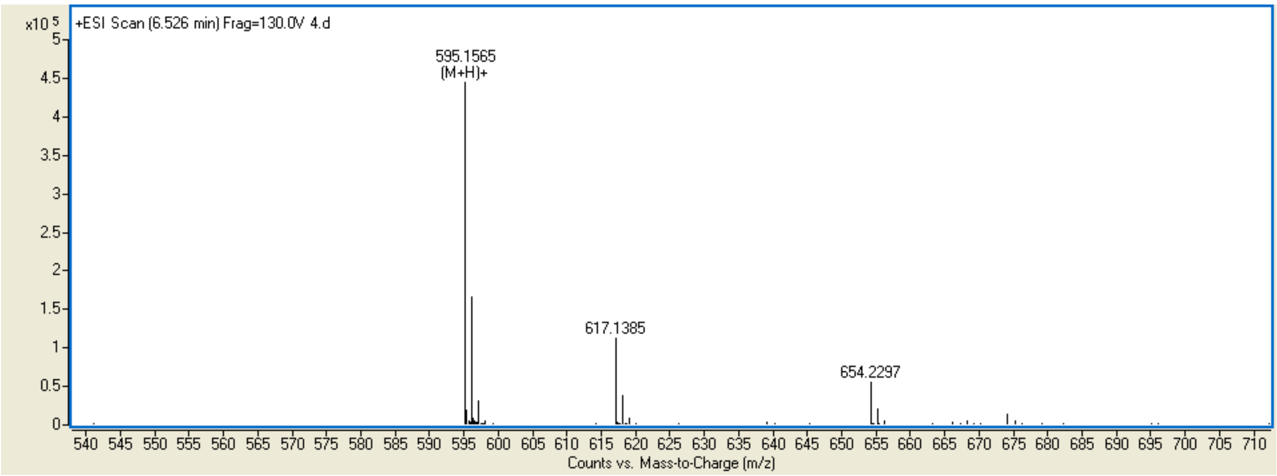

Figure S35. MS spectra of compound **4i**.

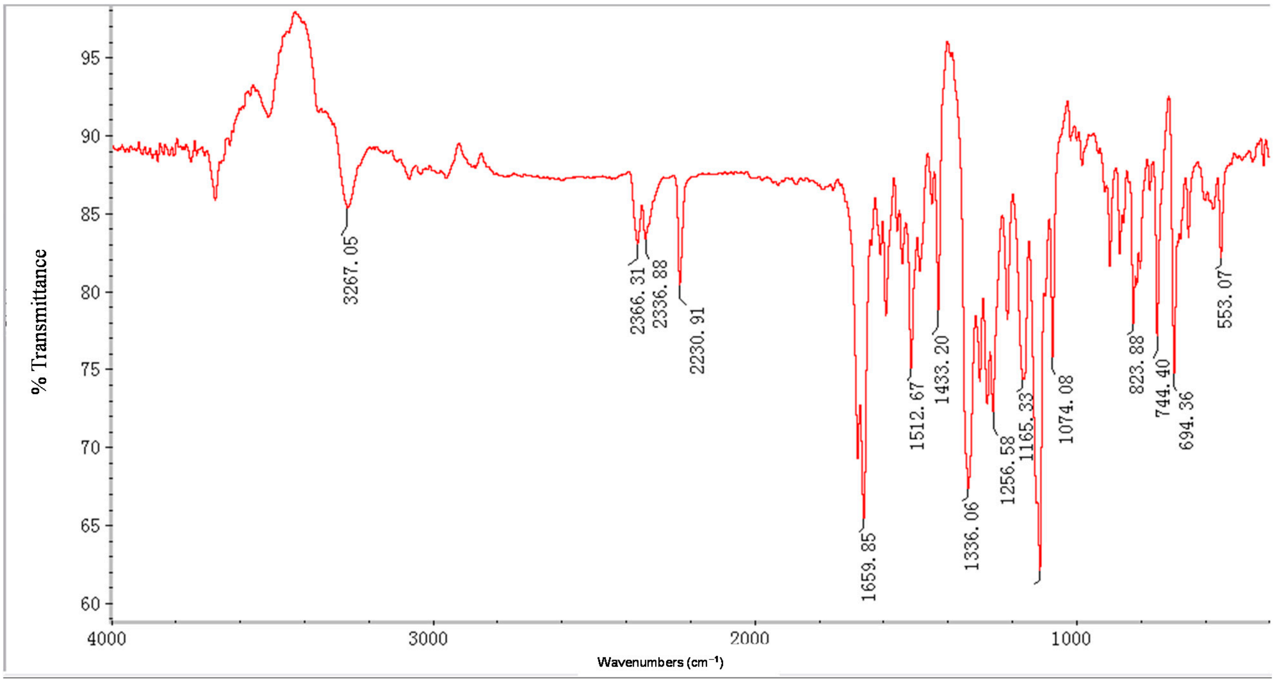

Figure S36. IR spectra of compound **4i**.

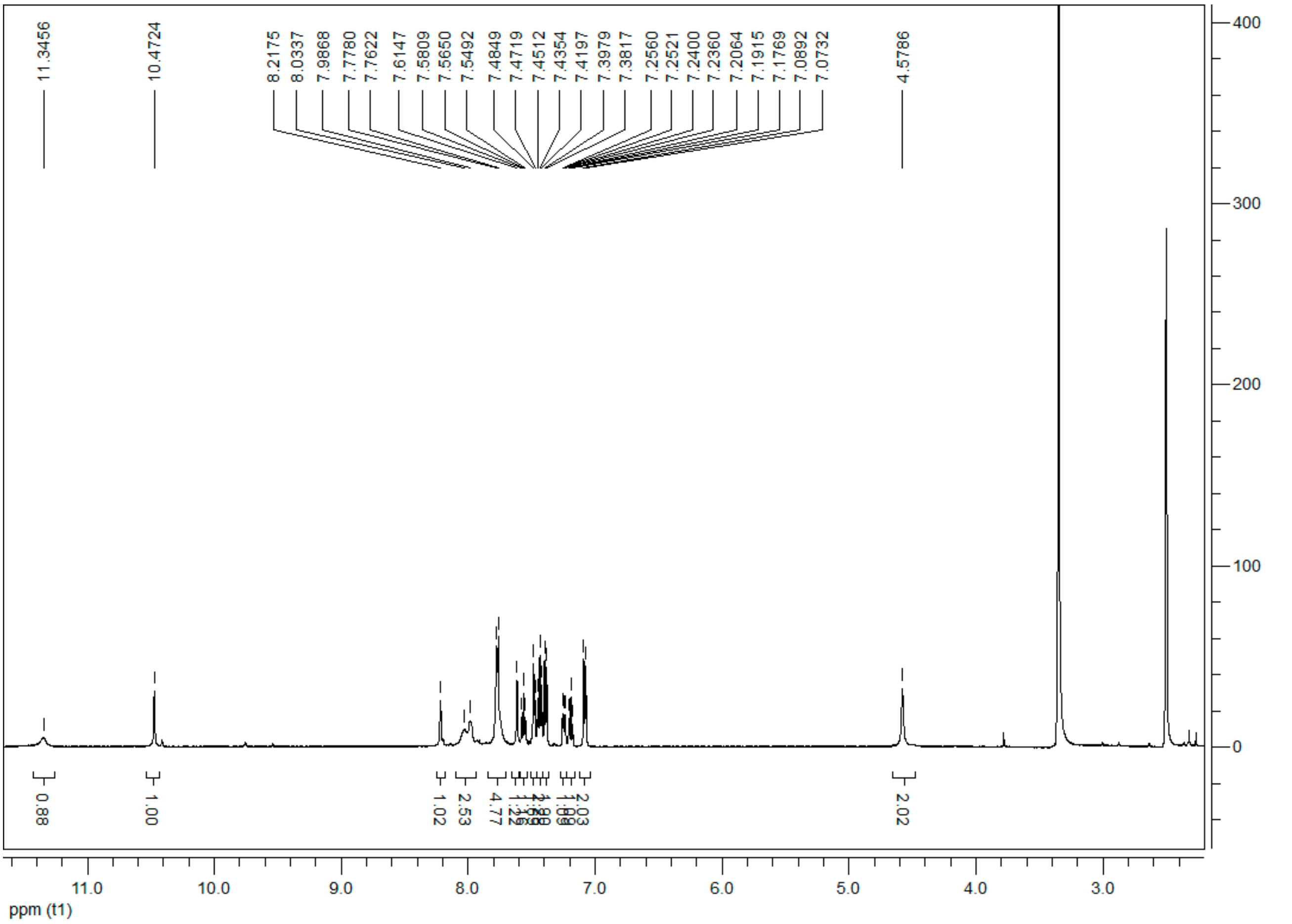

Figure S37. ^{1}H-NMR spectra of compound **4j**.

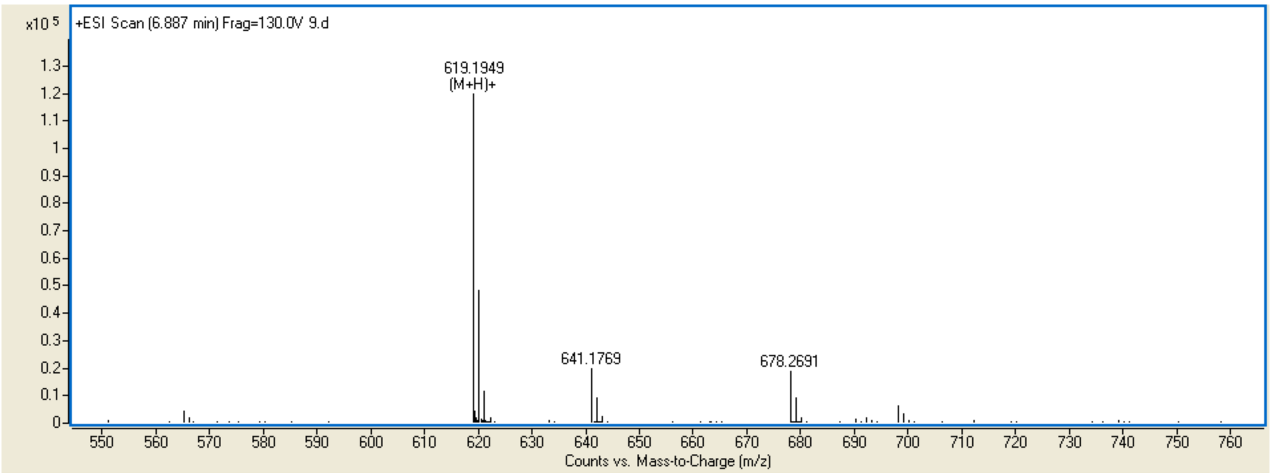

Figure S38. MS of compound **4j**.

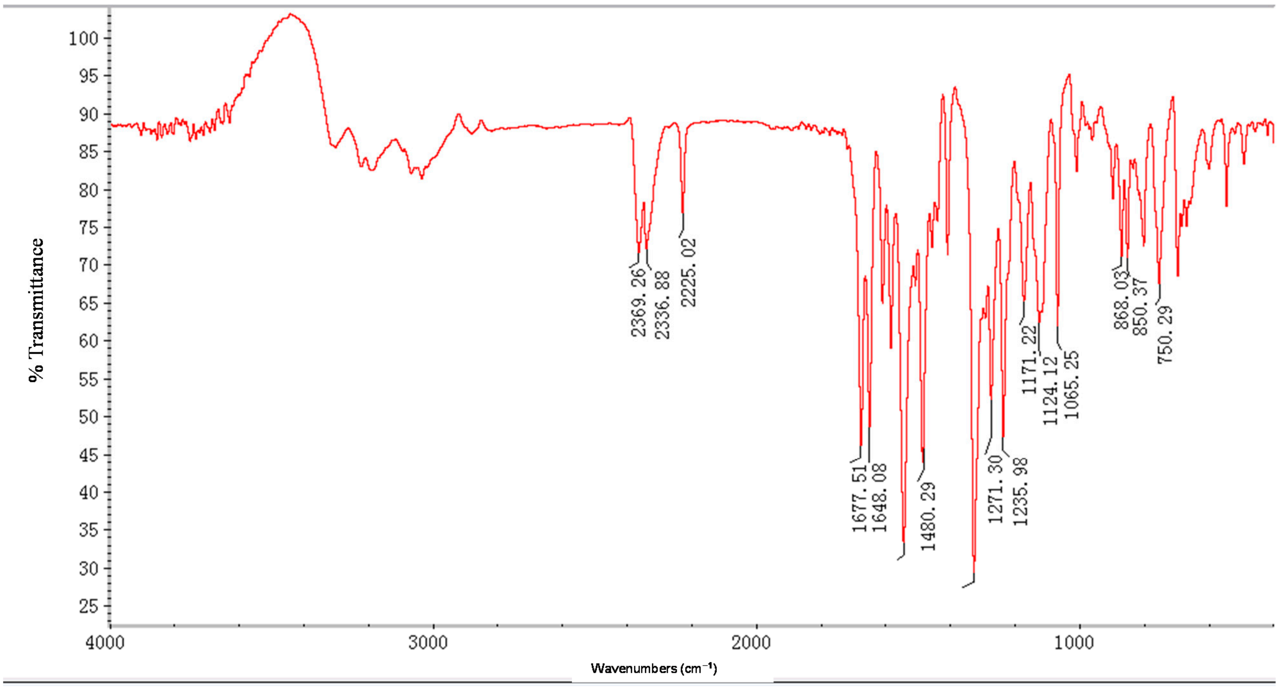

Figure S39. IR of compound **4j**.

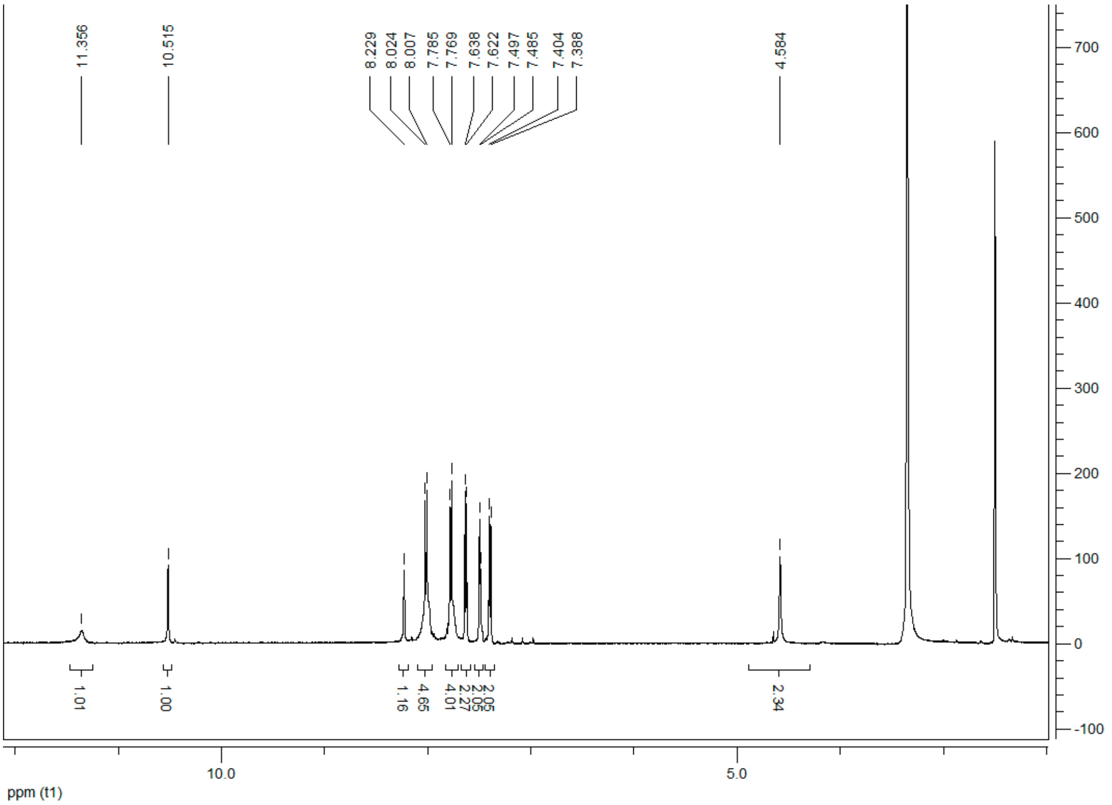

Figure S40. ^{1}H-NMR spectra of compound **4k**.

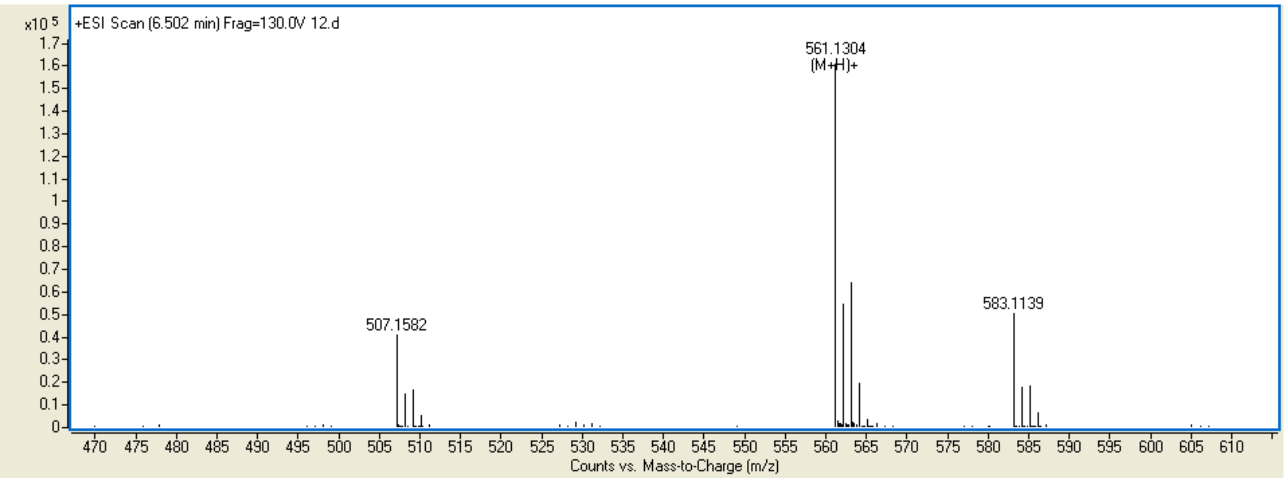

Figure S41. MS of compound **4k** w201312111.

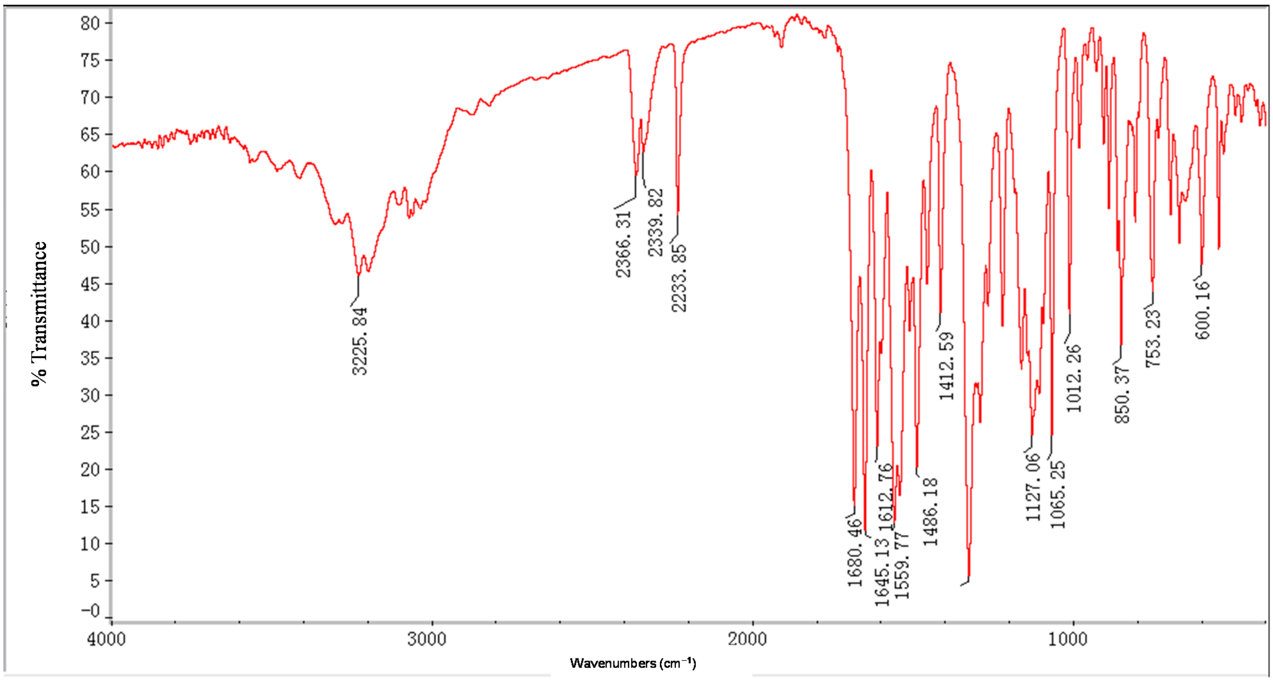

Figure S42. IR of compound **4k**.

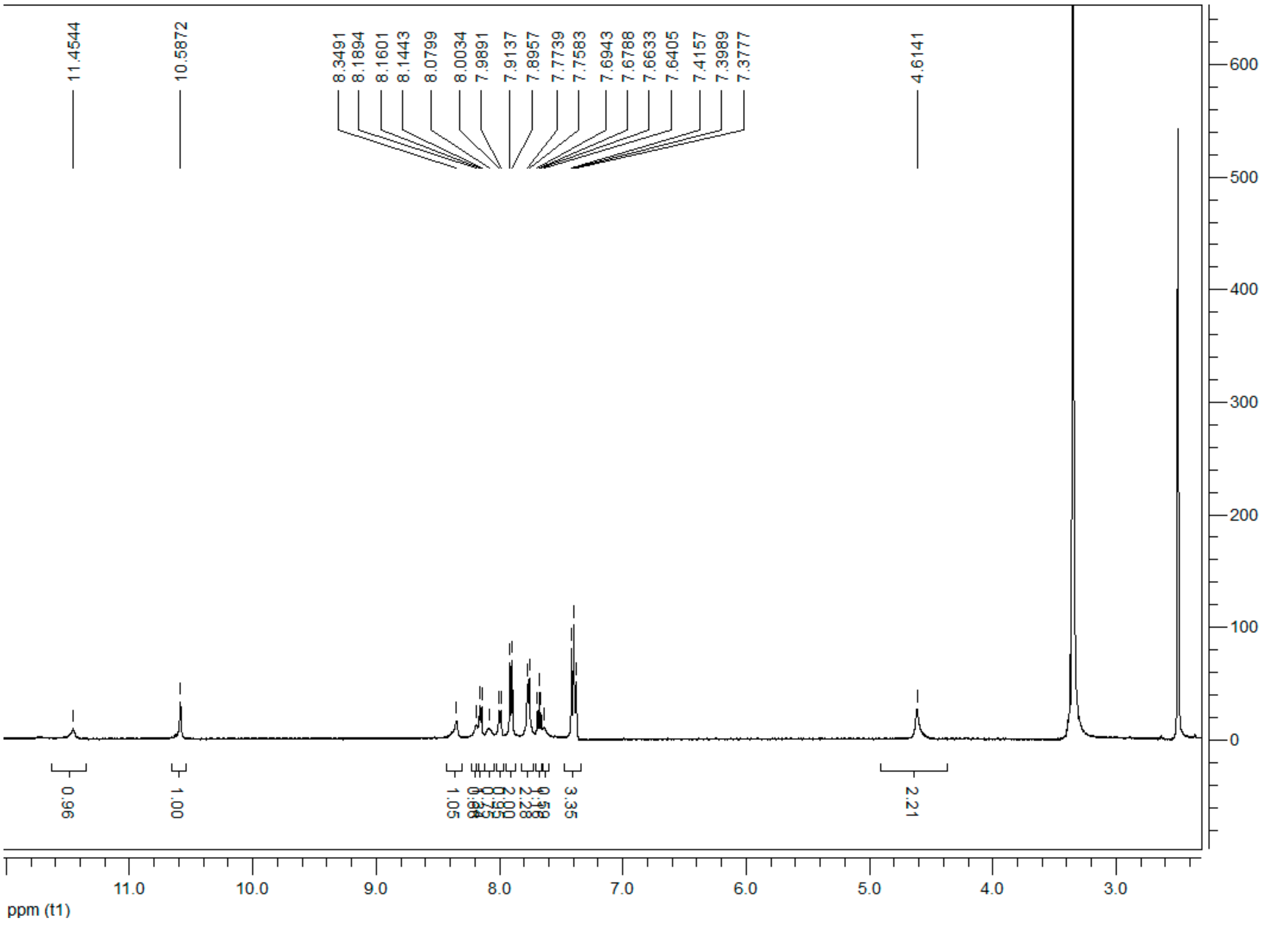

Figure S43. ^{1}H-NMR spectra of **4l**.

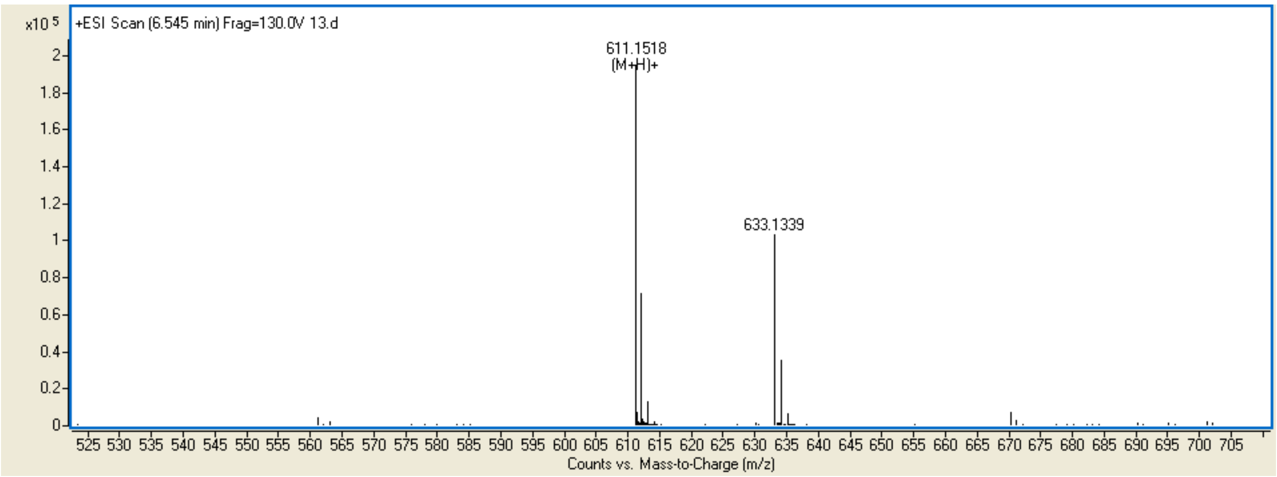

Figure S44. MS of compound **4l**.

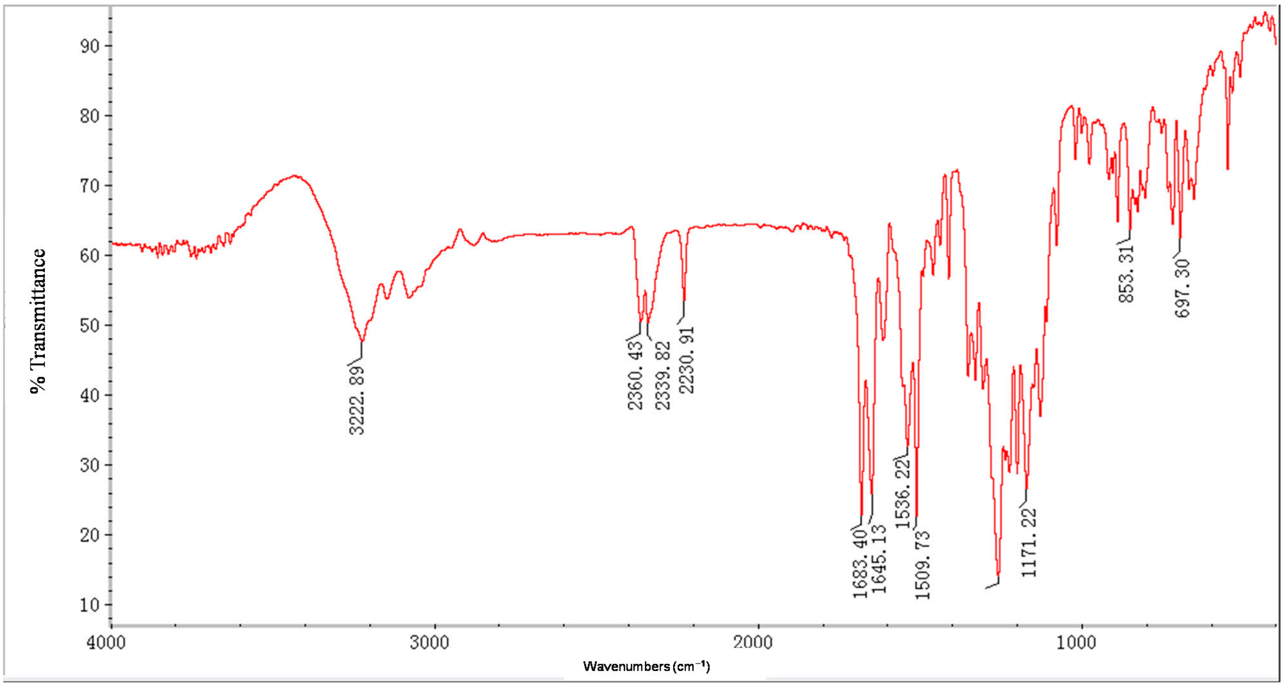

Figure S45. IR of compound **4l**.
